# Stabilizing ruthenium dioxide with cation-anchored sulfate for durable oxygen evolution in proton-exchange membrane water electrolyzers

**DOI:** 10.1038/s41467-023-43977-7

**Published:** 2023-12-07

**Authors:** Yanrong Xue, Jiwu Zhao, Liang Huang, Ying-Rui Lu, Abdul Malek, Ge Gao, Zhongbin Zhuang, Dingsheng Wang, Cafer T. Yavuz, Xu Lu

**Affiliations:** 1 CCRC, Division of Physical Science and Engineering (PSE), King Abdullah University of Science and Technology (KAUST), Thuwal, 23955-6900 Kingdom of Saudi Arabia; 2 KAUST Solar Center (KSC), PSE, KAUST, Thuwal, Kingdom of Saudi Arabia; 3 National Synchrotron Radiation Research Center, Hsinchu, 300 Taiwan; 4 State Key Lab of Organic-Inorganic Composites, Beijing University of Chemical Technology, 100029 Beijing, China; 5 Department of Chemistry, Tsinghua University, 100084 Beijing, China; 6 Advanced Membranes and Porous Materials Center (AMPM), PSE, KAUST, Thuwal, Kingdom of Saudi Arabia

**Keywords:** Electrocatalysis, Hydrogen fuel

## Abstract

Ruthenium dioxide is the most promising alternative to the prevailing but expensive iridium-based catalysts for the oxygen evolution reaction in proton-exchange membrane water electrolyzers. However, the under-coordinated lattice oxygen of ruthenium dioxide is prone to over-oxidation, and oxygen vacancies are formed at high oxidation potentials under acidic corrosive conditions. Consequently, ruthenium atoms adjacent to oxygen vacancies are oxidized into soluble high-valence derivatives, causing the collapse of the ruthenium dioxide crystal structure and leading to its poor stability. Here, we report an oxyanion protection strategy to prevent the formation of oxygen vacancies on the ruthenium dioxide surface by forming coordination-saturated lattice oxygen. Combining density functional theory calculations, electrochemical measurements, and a suite of *operando* spectroscopies, we showcase that barium-anchored sulfate can greatly impede ruthenium loss and extend the lifetime of ruthenium-based catalysts during acidic oxygen evolution, while maintaining the activity. This work paves a new way for designing stable and active anode catalysts toward acidic water splitting.

## Abstract (web summary)

Designing stable Ru-based catalysts for acidic oxygen evolution remains a challenge. Here, the authors propose an oxyanion protection strategy to prevent the formation of oxygen vacancies on the RuO_2_ surface by creating coordination-saturated lattice oxygen, thereby greatly enhancing the stability.

## Introduction

Hydrogen (H_2_) production by water electrolysis, especially when powered by renewable energy, represents a promising and sustainable approach to mitigating carbon emissions while meeting the energy demand^1,2^. Among incumbent water electrolyzers, the proton-exchange membrane water electrolyzer (PEMWE) has exhibited the advantages of compact reactor design, high operating current density, and rapid response^3,4^. PEMWE is even more appealing to the industry in light of the high pressure (3.0–7.6 MPa) and high purity (>99.9999 vol%) of its produced H_2_^5^. However, the wide deployment of PEMWEs has been greatly limited by the anode oxygen evolution reaction (OER) catalyst, which can be easily deactivated at high-oxidation potentials under acidic corrosive conditions^6^.

Iridium (Ir) and its derived materials have shown practical OER stability in acid, whereby becoming the prevailing anode catalysts for PEMWEs^7^. Yet, the large-scale application of Ir-based PEMWEs has been largely hindered by its high cost^8^. Thus far, major advances have been reported in reducing the anode cost of PEMWE: (1) developing high-performance Ir-based catalysts with low Ir usage;^9,10^ and (2) exploring cost-effective alternative materials to Ir that meet the requirements of PEMWE applications^11,12^. Ruthenium dioxide (RuO_2_)—with a much lower price, higher OER activity, and lower noble metal loading—is considered a viable alternative to Ir for PEMWEs^13^. However, PEMWE stacks are usually operated at current densities exceeding 500 mA cm^−2^ ^14^. Higher reactivity, with its concomitant faster proton transfer, acidifies the immediate vicinity of the PEM. Consequently, the acidity in close proximity to the anode is stronger than 1 M H_2_SO_4_ when using distilled water as the electrolyte, and this effect is exacerbated when using acidic electrolytes or high current densities^15^. To date, the stability of Ru-based OER catalysts has remained poor under such harsh acidic corrosive and oxidative conditions^16^.

Mechanistically, the poor stability of RuO_2_ during acidic OER is caused by the over-oxidation of its lattice oxygen (O) to form O vacancies (V_o_)^6,13^. As a consequence, the Ru atoms adjacent to V_o_ are exposed to the electrolyte and become susceptible to being over-oxidized into soluble high-valence Ru^x>4^ derivatives (e.g., RuO_4_), leading to the RuO_2_ crystal structure collapse and surface Ru loss^17^. Thus far, two strategies have been proposed to improve the stability of RuO_2_-based electrocatalysts: (1) Enhancing the intrinsic acidic OER activity to lower the required overpotential at controlled water-splitting current, thereby mitigating the catalyst degradation^18,19^. To this end, extensive efforts have been devoted to increasing the number of active sites and adjusting the adsorption energy of oxygenated intermediate species (*OH, *O, *OOH, etc.)^20,21^, including morphology design, electronic structure reconfiguration, strain regulation, defect construction, single-atom tuning, and heterostructure engineering^22,23^. (2) Stabilizing the lattice O to reduce the number of V_o_ that can be formed during acidic OER^16,24^. Ru-based high entropy oxides or multimetal oxides could form strong metal-O (M-O) bonds to suppress the over-oxidation of lattice O^25,26^. For example, Zhang et al. proposed the co-doping of tungsten (W) and erbium (Er) into the lattice of RuO_2_, which enhanced the acidic OER stability to some extent^17^. However, the field has mainly focused on using metal cations to modify Ru-based catalysts^27^. To date, the role of anions in stabilizing Ru-based materials under acidic conditions has remained unexplored.

In this work, we revealed the RuO_2_ deactivation mechanism during acidic OER using density functional theory (DFT), suggesting that the lattice O atoms on the crystal grain edges of RuO_2_ are more prone to over-oxidation than those on the lattice planes due to the unsaturated coordination. Guided by the DFT predictions, we designed an oxyanion protection strategy to prevent the formation of V_o_ on the RuO_2_ surface by forming coordination-saturated lattice O. According to the binding energy of various metal cations with anions when coordinated with RuO_2_, we screened out barium (Ba) anchored sulfate to protect the lattice O, and simultaneously incorporated W to lower the OER overpotential. The as-prepared Ba_0.3_(SO_4_)_*δ*_W_0.2_Ru_0.5_O_2−*δ*_ catalyst delivered a current density of 10 mA cm^−2^ at a low overpotential of 206 mV with >1000 h stability in 0.5 M H_2_SO_4_, significantly outperforming commercial RuO_2_. The enhanced acidic OER performance was then investigated by DFT calculations and a series of *operando* spectroscopies, ascertaining the functions of (1) Ba in retaining sulfate on the catalyst surface, (2) sulfate (anchored by Ba) in preventing the over-oxidation of lattice O while weakening the adsorption energy of oxygenated intermediates and switching the rate-determining step (RDS), and (3) W in accelerating the altered RDS—that is, the dissociation of chemisorbed water to form *OOH. The protection of lattice O by Ba-anchored sulfate and the role of W in lowering the overpotential collectively resulted in exceptional acidic OER stability. More remarkably, the Ba_0.3_(SO_4_)_*δ*_W_0.2_Ru_0.5_O_2−*δ*_ catalyst, when assembled in a PEMWE, performed stable OER in 0.5 M H_2_SO_4_ for 300 h at a water-splitting current density of 500 mA cm^−2^, certifying the effectiveness of the proposed RuO_2_ stabilizing strategies in practical applications.

## Results

### Theory-guided RuO_2_ stabilizing strategy

**Fig. 1:**
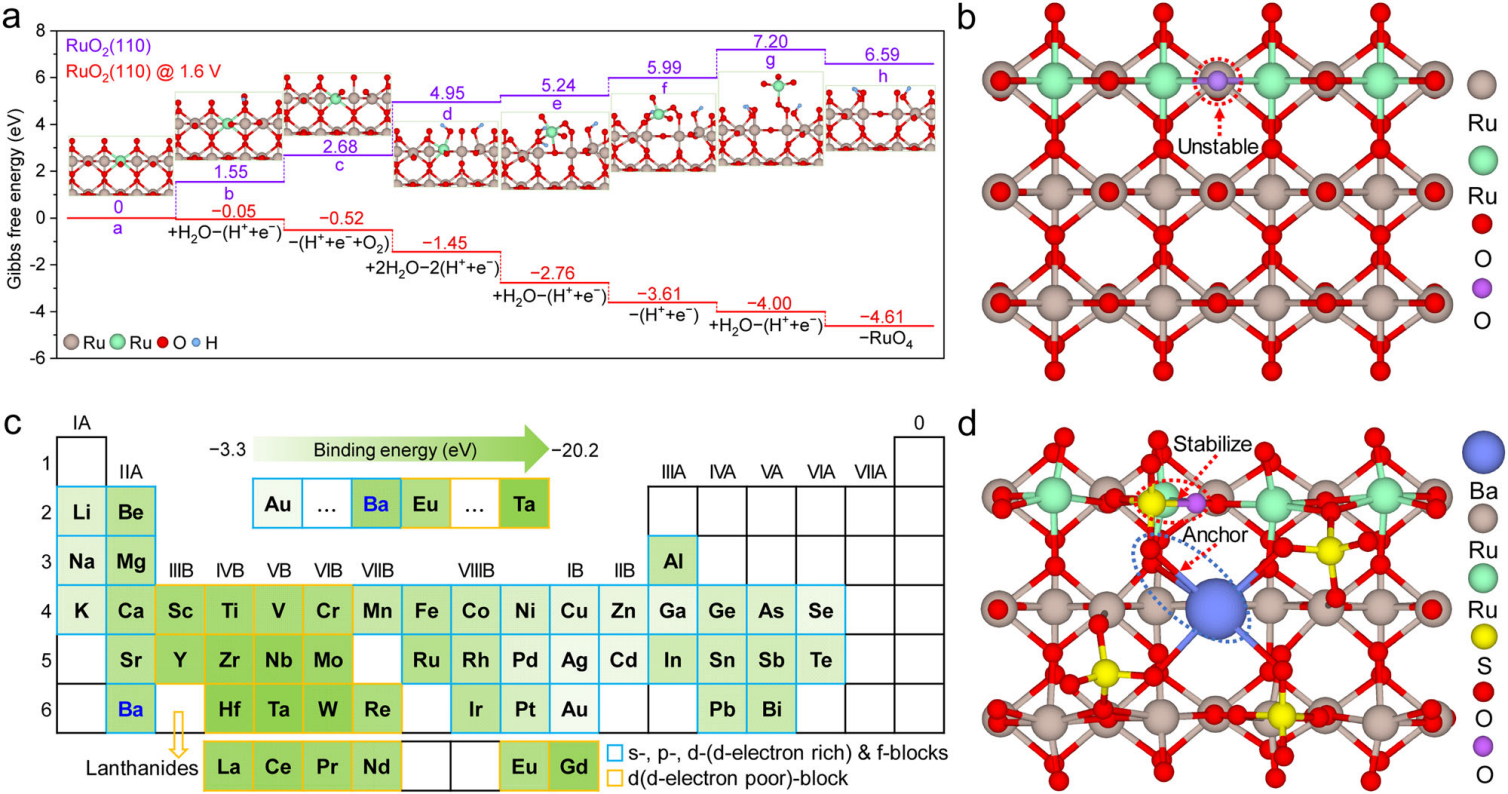
RuO_2_ stabilizing strategy guided by density functional theory. **a** Gibbs free energy diagram of Ru dissolution on the RuO_2_ (110) plane. Of note, the green ball represents the Ru atom on the grain edge. **b** Ball-and-stick model of the RuO_2_ (110) plane (see Supplementary Fig. 2 for another viewing angle). Of note, the green and purple balls represent Ru and O atoms, respectively, on the grain edges. **c** Binding energy of various metal elements with sulfate on the RuO_2_ (110) plane, and the intensity of green color represents the strength of the binding energy for each metal element (Supplementary Table 1). Of note, “*d*-block metals with poor *d*-electrons” refers to metals with *d* orbital electrons ≤5, and “*d*-electron rich” refers to metals with *d* orbital electrons ≥5. **d** Ball-and-stick model of Ba-anchored sulfate on the RuO_2_ (110) plane (see Supplementary Fig. 4 for another viewing angle). Of note, the green and purple balls represent Ru and O atoms, respectively, on the grain edges.

We first developed a DFT framework to investigate the deactivation mechanism of RuO_2_ during acidic OER (Fig. 1a). RuO_2_ (110), a well-characterized crystal plane, was selected for the calculation^28^. In the outermost layer of the RuO_2_ (110) plane, there are two types of under-coordinated surface atoms^13,29^: (1) a lattice O located on the surface or the edge of the plane and coordinated to two (i.e., bridging O, instead of three in bulk) Ru atoms, and (2) a one-fold coordinatively unsaturated Ru sites (1f-cus Ru) coordinated to five (instead of six in bulk) O atoms. Compared to the relatively stable crystal planes, the grain edges are metastable due to anisotropy and coordinative unsaturation^30,31^, rendering them more likely to incur electrochemical/chemical corrosion^32^. Consistent with this, our DFT results showed that at an oxidation potential of 1.6 V (vs. reversible hydrogen electrode, RHE, unless otherwise stated), it is energetically favorable for the 1f-cus Ru active sites on the grain edges to undergo the adsorption of water and deprotonation, and eventually become soluble RuO_4_ (Supplementary Fig. 1), suggesting that the lattice O near the 1f-cus Ru active sites on the grain edges are more prone to overoxidation than those in bulk or on the surface of the catalyst (Fig. 1b and Supplementary Fig. 2)^33^. Therefore, stabilizing lattice O on the grain edges of RuO_2_ can drastically improve its stability.

Oxyanions with under-coordinated O atoms tend to bond with 1f-cus Ru to form saturated sites, thereby stabilizing the lattice O bond to Ru on the RuO_2_ surface. Sulfate, which fits within the RuO_2_ (110) facet (Supplementary Fig. 3), may well fulfill this purpose. However, sulfate is vulnerable to acidic and oxidative conditions^34,35^. We therefore envisaged fixing metal cations (M) in Ru lattice sites to coordinate with the O atoms in sulfate, thus immobilizing sulfate on the crystal. Screening of M across the periodic table was then performed based on their chemical binding energies (Supplementary Table 1). Despite having superior binding energy with sulfate, *d*-block metals with poor *d*-electrons were first ruled out because they are chemically active and intrinsically unstable (Fig. 1c)^13^. Among the remaining metal elements in the *s-*, *p-*, *d (d-electron rich)-*, and *f-*blocks, Ba possesses the strongest binding energy with sulfate on the RuO_2_ (110) plane. Furthermore, the sulfate coordinated with Ba matches well with RuO_2_ (110) structurally, where the coordination-saturated O shared by sulfate and Ru is located on the RuO_2_ lattice site, and Ba fixes sulfate by bonding with one of its O atoms (Fig. 1d and Supplementary Fig. 4). Therefore, we selected Ba-anchored sulfate to stabilize the RuO_2_.

### Synthesis and characterization of Ba_0.3_(SO_4_)_*δ*_W_0.2_Ru_0.5_O_2−*δ*_ and control samples

**Fig. 2:**
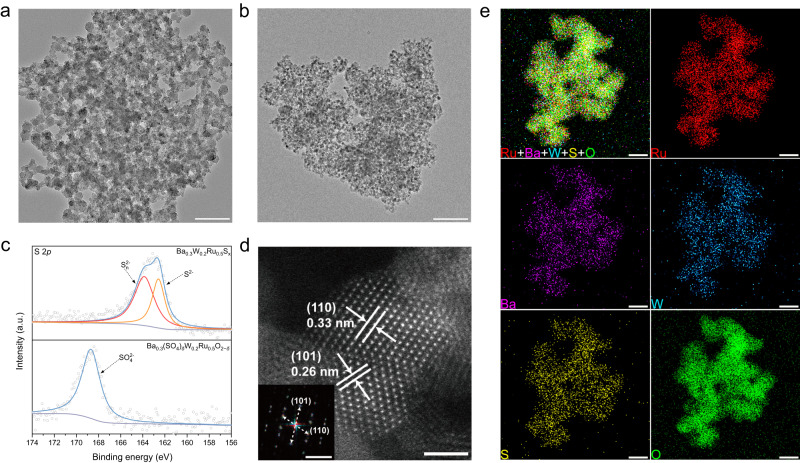
Preparation and characterization of the catalysts. **a**, **b** TEM images of **a** Ba_0.3_W_0.2_Ru_0.5_S_x_ and **b** Ba_0.3_(SO_4_)_*δ*_W_0.2_Ru_0.5_O_2−*δ*_ (scale bar, 100 nm). **c** High-resolution XPS spectra of S 2*p* for Ba_0.3_W_0.2_Ru_0.5_S_x_ and Ba_0.3_(SO_4_)_*δ*_W_0.2_Ru_0.5_O_2−*δ*_. **d** HAADF–STEM image of Ba_0.3_(SO_4_)_*δ*_W_0.2_Ru_0.5_O_2−*δ*_ (scale bar, 2 nm). Inset: FFT analysis of Ba_0.3_(SO_4_)_*δ*_W_0.2_Ru_0.5_O_2−*δ*_ (scale bar, 5 nm^−1^). **e** EDS mapping of Ba_0.3_(SO_4_)_*δ*_W_0.2_Ru_0.5_O_2−*δ*_ (scale bar, 20 nm).

The theoretical predictions guided us to incorporate Ba-anchored sulfate into RuO_2_ to stabilize the catalyst. W dopant was also introduced into the synthesis process to enhance the OER activity (Supplementary Fig. 5). First, metal precursors and sulfur (S) sources were dissolved in an oleylamine solution, which was heated to 250 °C to obtain Ba_0.3_W_0.2_Ru_0.5_S_x_ nanosheets (Fig. 2a). Then, the Ba_0.3_W_0.2_Ru_0.5_S_x_ nanosheets were loaded on carbon black, followed by calcination at 400 °C in an air atmosphere. Finally, the as-obtained material was acid-washed to remove impurities, forming Ba_0.3_(SO_4_)_*δ*_W_0.2_Ru_0.5_O_2−*δ*_ nanoparticles (Fig. 2b). Figure 2c shows the S 2*p* X-ray photoelectron spectroscopy (XPS) spectra of Ba_0.3_W_0.2_Ru_0.5_S_x_ and Ba_0.3_(SO_4_)_*δ*_W_0.2_Ru_0.5_O_2−*δ*_. The peaks at 162.6 and 163.9 eV were assigned to sulfide (S^2−^) and polysulfides (S_n_^2−^) species in Ba_0.3_W_0.2_Ru_0.5_S_x_, respectively^36^. In contrast, only one peak was observed at 168.7 eV for Ba_0.3_(SO_4_)_*δ*_W_0.2_Ru_0.5_O_2−*δ*_, which was ascribed to SO_4_^2−^ (see ref. ^37^), suggesting that the sulfide was oxidized to sulfate after calcination. The X-ray diffraction (XRD) pattern of Ba_0.3_(SO_4_)_*δ*_W_0.2_Ru_0.5_O_2−*δ*_ presented the characteristic peaks of rutile RuO_2_ (JCPDS card No. 88-0286), confirming the formation of oxides (Supplementary Fig. 6). High-angle annular dark-field scanning transmission electron microscopy (HAADF–STEM) of Ba_0.3_(SO_4_)_*δ*_W_0.2_Ru_0.5_O_2−*δ*_ indicated well-defined lattice fringes with interplanar distances of 0.33 and 0.26 nm for the (110) and (101) planes of rutile-structured RuO_2_, respectively (Fig. 2d). Consistent with the HAADF–STEM image, fast Fourier transform (FFT) analysis and selected area electron diffraction (SAED) pattern showed distinct diffraction rings of RuO_2_ (inset in Fig. 2d and Supplementary Fig. 7). Energy-dispersive X-ray spectrometry (EDS) elemental mapping of Ba_0.3_(SO_4_)_*δ*_W_0.2_Ru_0.5_O_2−*δ*_ revealed the homogeneous distribution of Ru, W, Ba, S, and O in the particles (Fig. 2e). Inductively coupled plasma mass spectrometry (ICP-MS) determined the mass fractions of Ru, W, Ba, and S in Ba_0.3_(SO_4_)_*δ*_W_0.2_Ru_0.5_O_2−*δ*_ to be ~36, 28, 27, and 9%, respectively. For comparison, Ba_m_(SO_4_)_*δ*_W_n_Ru_1−m−n_O_2−*δ*_ with various element ratios, and a series of Ru-based catalysts, including RuO_2_ (named “as-prepared RuO_2_”), W_0.3_Ru_0.7_O_2_, Ba_0.4_Ru_0.6_O_2_, (SO_4_)_*δ*_RuO_2−*δ*_, Ba_0.4_(SO_4_)_*δ*_Ru_0.6_O_2−*δ*_, and W_0.3_(SO_4_)_*δ*_Ru_0.7_O_2−*δ*_, were synthesized through a similar process (Supplementary Figs. 8 and 9).

### Acidic OER performance of the catalysts

**Fig. 3:**
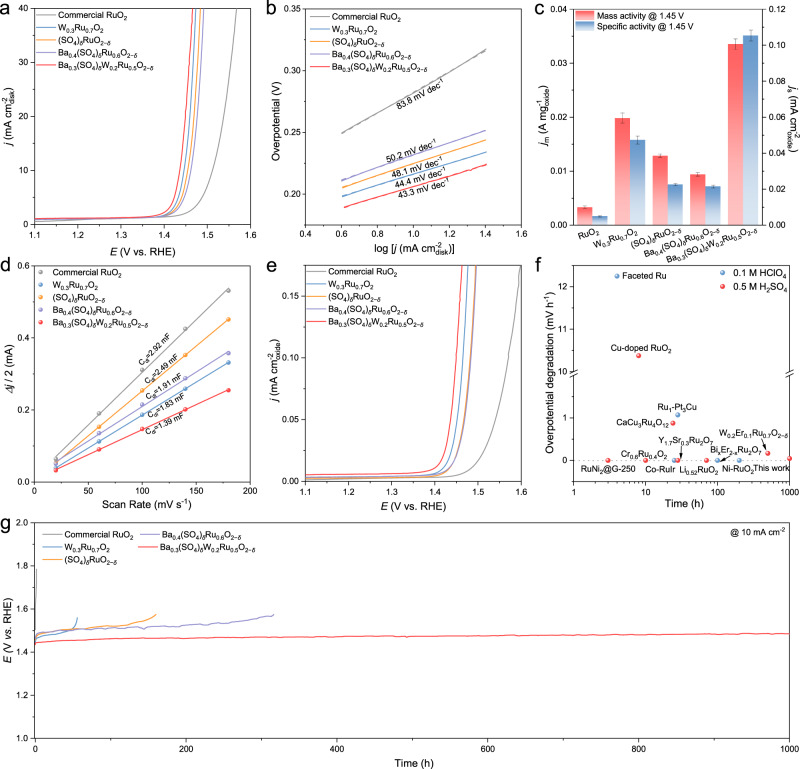
OER performance of catalysts in 0.5 M H_2_SO_4_. **a** OER polarization curves, **b** Tafel plots, **c** Mass and specific activity at 1.45 V with error bar, **d** C_dl_ plots at 1.25 V, and **e** ECSA-normalized polarization curves of commercial RuO_2_, W_0.3_Ru_0.7_O_2_, (SO_4_)_*δ*_RuO_2−*δ*_, Ba_0.4_(SO_4_)_*δ*_Ru_0.6_O_2−*δ*_, and Ba_0.3_(SO_4_)_*δ*_W_0.2_Ru_0.5_O_2−*δ*_. Of note, the catalyst loading is 125 μg_oxide_ cm^−2^, and the area of the glassy carbon electrode is 0.196 cm^2^. **f** Summary of the acidic OER stability of up-to-date Ru-based catalysts at 10 mA cm^−2^ (Supplementary Table 5). **g** Chronopotentiogram of commercial RuO_2_, W_0.3_Ru_0.7_O_2_, (SO_4_)_*δ*_RuO_2−*δ*_, Ba_0.4_(SO_4_)_*δ*_Ru_0.6_O_2−*δ*_, and Ba_0.3_(SO_4_)_*δ*_W_0.2_Ru_0.5_O_2−*δ*_ at 10 mA cm^−2^ in 0.5 M H_2_SO_4_.

The acidic OER performance of Ba_0.3_(SO_4_)_*δ*_W_0.2_Ru_0.5_O_2−*δ*_ was evaluated on a rotating disk electrode (RDE) in a typical three-electrode electrolytic cell, and the electrolyte was 0.5 M H_2_SO_4_ unless otherwise stated. Control samples such as RuO_2_, W_0.3_Ru_0.7_O_2_, (SO_4_)_*δ*_RuO_2−*δ*_, and Ba_0.4_(SO_4_)_*δ*_Ru_0.6_O_2−*δ*_ were tested under the same conditions. All the samples were measured based on three independent tests (Supplementary Fig. 10). Compared to commercial RuO_2_, the as-prepared RuO_2_ exhibits poor OER performance (Supplementary Fig. 11), so we use commercial RuO_2_ as the benchmark. As displayed in Fig. 3a, the OER activities of the prepared catalysts followed an order of Ba_0.3_(SO_4_)_*δ*_W_0.2_Ru_0.5_O_2−*δ*_ > W_0.3_Ru_0.7_O_2_ > (SO_4_)_*δ*_RuO_2−*δ*_ > Ba_0.4_(SO_4_)_*δ*_Ru_0.6_O_2−*δ*_ > commercial RuO_2_. The Ba_0.3_(SO_4_)_*δ*_W_0.2_Ru_0.5_O_2−*δ*_ catalyst exhibited an onset potential of ~1.35 V and delivered a current density of 10 mA cm^−2^ at an overpotential of 206 mV, lower than of commercial RuO_2_ at 282 mV (Supplementary Table 2). Ba_0.3_(SO_4_)_*δ*_W_0.2_Ru_0.5_O_2−*δ*_ also showed the highest OER activity among the Ba_m_(SO_4_)_*δ*_W_n_Ru_1−m−n_O_2−*δ*_ catalysts (Supplementary Fig. 12). Furthermore, the Tafel slope decreased from 83.8 mV dec^−1^ on commercial RuO_2_ to 43.3 mV dec^−1^ on Ba_0.3_(SO_4_)_*δ*_W_0.2_Ru_0.5_O_2−*δ*_, suggesting that Ba_0.3_(SO_4_)_*δ*_W_0.2_Ru_0.5_O_2−*δ*_ can more effectively lower the acidic OER overpotential than commercial RuO_2_ at higher current densities (Fig. 3b)^17,38^. According to the electrochemical impedance spectroscopy (EIS) results at 1.5 V, Ba_0.3_(SO_4_)_*δ*_W_0.2_Ru_0.5_O_2−*δ*_ achieved the lowest charge-transfer resistance (R_ct_) of 2.83 Ω among all tested catalysts, implying its enhanced OER kinetics (Supplementary Fig. 13)^19^.

To better understand the effects of the incorporated W and sulfate anchored by Ba on acidic OER activity, the mass activity (*j*_m_) of the prepared catalysts was calculated at 1.45 V (Fig. 3c). Ba_0.3_(SO_4_)_*δ*_W_0.2_Ru_0.5_O_2−*δ*_ exhibited the highest metal oxide-based mass activity of 0.034 A mg_oxide_^−1^, over 11.3 times as high as that of commercial RuO_2_ (0.003 A mg_oxide_^−1^). The specific activity (*j*_s_) of the catalysts at 1.45 V was determined by measuring their electrochemical double-layer capacitance (C_dl_) and calculating the electrochemically active surface area (ECSA) (Supplementary Fig. 14)^20^. Ba_0.3_(SO_4_)_*δ*_W_0.2_Ru_0.5_O_2−*δ*_ with large nanoparticles delivered the lowest C_dl_ (1.39 mF), less than half that of RuO_2_ (2.92 mF) (Fig. 3d). The ECSA-normalized OER activities of the catalysts followed the order of Ba_0.3_(SO_4_)_*δ*_W_0.2_Ru_0.5_O_2−*δ*_ > W_0.3_Ru_0.7_O_2_ > (SO_4_)_*δ*_RuO_2−*δ*_ ≈ Ba_0.4_(SO_4_)_*δ*_Ru_0.6_O_2−*δ*_ > commercial RuO_2_ (Fig. 3e), and the *j*_s_ of Ba_0.3_(SO_4_)_*δ*_W_0.2_Ru_0.5_O_2−*δ*_ (0.105 mA cm_oxide_^−2^) was 21-fold higher than that of RuO_2_ (0.005 mA cm_oxide_^−2^). Furthermore, we also calculated the areal mass activity of the catalysts at 1.45 V, and the areal mass activity of Ba_0.3_(SO_4_)_*δ*_W_0.2_Ru_0.5_O_2−*δ*_ (0.473 A mg_Ru_^−1^ cm_disk_^−2^) was 21.5-fold higher than that of RuO_2_ (0.022 A mg_Ru_^−1^ cm_disk_^−2^) (Supplementary Fig. 15). These results indicated that the intrinsic OER activity of each active site was enhanced on Ba_0.3_(SO_4_)_*δ*_W_0.2_Ru_0.5_O_2−*δ*_. Of note, the similar specific activities of (SO_4_)_*δ*_RuO_2−*δ*_ and Ba_0.4_(SO_4_)_*δ*_Ru_0.6_O_2−*δ*_, and the comparable OER activities of Ba_0.4_Ru_0.6_O_2_ and commercial RuO_2_ implied the negligible contribution of Ba to boosting the OER activity (Supplementary Fig. 16).

Next, the catalyst durability was assessed using the chronopotentiometry (CP) method at 10 mA cm^−2^ in 0.5 M H_2_SO_4_ (Fig. 3g). The OER performance of commercial RuO_2_ drastically degraded within 1.5 h, while W_0.3_Ru_0.7_O_2_ lasted for 55 h. This indicated that introducing W can improve the stability of RuO_2_, likely because of the lowered OER overpotential, albeit to a limited extent. In comparison, doping sulfate can better stabilize RuO_2_, improving the stability to 160 h on (SO_4_)_*δ*_RuO_2−*δ*_, although sulfate losses eventually occurred. To measure the amount of dissolved S, (SO_4_)_*δ*_RuO_2−*δ*_ was tested at 10 mA cm^−2^ for 100 h in 0.1 M HClO_4_ (Supplementary Fig. 17), and around 64.1% of S was dissolved into the electrolyte as suggested by ICP-MS. In line with our theoretical predictions, incorporating Ba could fix the sulfate on the RuO_2_ surface – only 25.6% of S in Ba_0.4_(SO_4_)_*δ*_Ru_0.6_O_2−*δ*_ was dissolved into the electrolyte under the same testing conditions. As a result, the OER durability of Ba_0.4_(SO_4_)_*δ*_Ru_0.6_O_2−*δ*_ at 10 mA cm^−2^ in 0.5 M H_2_SO_4_ was further extended to 316 h. In comparison, the OER activity and stability of W_0.3_(SO_4_)_*δ*_Ru_0.7_O_2−*δ*_ are slightly better than those of (SO_4_)_*δ*_RuO_2−*δ*_, indicating that the W dopant can lower the OER overpotential rather than immobilize sulfate (Supplementary Fig. 18). By synergizing the stability-enhancing effects of W and sulfate anchored by Ba, the Ba_0.3_(SO_4_)_*δ*_W_0.2_Ru_0.5_O_2−*δ*_ catalyst presented an overpotential degradation of only 43 mV after 1000 h continuous OER at 10 mA cm^−2^ in 0.5 M H_2_SO_4_. After analyzing the electrolyte after the OER stability test, Ba_0.3_(SO_4_)_*δ*_W_0.2_Ru_0.5_O_2−*δ*_ incurred the smallest Ru loss (7.1%) among all tested catalysts (Supplementary Table 3), and the stability number (S-number) of catalysts followed the order of Ba_0.3_(SO_4_)_*δ*_W_0.2_Ru_0.5_O_2−*δ*_ > Ba_0.4_(SO_4_)_*δ*_Ru_0.6_O_2−*δ*_ > (SO_4_)_*δ*_RuO_2−*δ*_ > W_0.3_Ru_0.7_O_2_ > commercial RuO_2_ (Supplementary Table 4). The notable durability of Ba_0.3_(SO_4_)_*δ*_W_0.2_Ru_0.5_O_2−*δ*_, with relatively low overpotential degradation and robust activity, surpassed up-to-date Ru-based catalysts under similar conditions (Fig. 3f and Supplementary Table 5). The prolonged acidic OER stability of our Ba_0.3_(SO_4_)_*δ*_W_0.2_Ru_0.5_O_2−*δ*_ catalyst was also verified by accelerated aging tests via fast-scan cyclic voltammetry (CV). Ba_0_._3_(SO_4_)_*δ*_W_0_._2_Ru_0_._5_O_2−*δ*_ showed only 28 mV overpotential increase at 10 mA cm^−2^ in 0.5 M H_2_SO_4_ after 100,000 cycles, whereas RuO_2_ was completely deactivated after 100,000 CV cycles (Supplementary Fig. 19).

### Underlying mechanism of enhanced acidic OER activity on Ba_0.3_(SO_4_)_*δ*_W_0.2_Ru_0.5_O_2−*δ*_

**Fig. 4:**
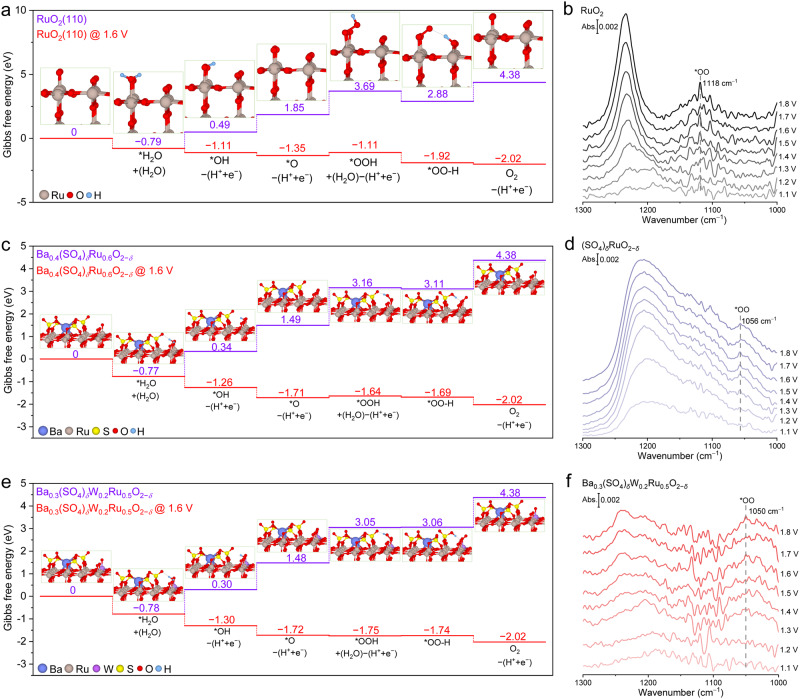
Mechanism of enhanced acidic OER activity on Ba_0.3_(SO_4_)_*δ*_W_0.2_Ru_0.5_O_2−*δ*_. **a**, **c**, **e** Gibbs free energy diagrams on the RuO_2_ (110) plane of (**a**) RuO_2_, **c** Ba_0.4_(SO_4_)_*δ*_Ru_0.6_O_2−*δ*_, and **e** Ba_0.3_(SO_4_)_*δ*_W_0.2_Ru_0.5_O_2−*δ*_ (see Supplementary Fig. 20 for detailed description). **b**, **d**, **f** In situ ATR-SEIRAS spectra acquired on **b** RuO_2_, **d** (SO_4_)_*δ*_RuO_2−*δ*_, and **f** Ba_0.3_(SO_4_)_*δ*_W_0.2_Ru_0.5_O_2−*δ*_ at acidic OER potentials from 1.1 to 1.8 V. Of note, the signal-to-noise ratio of our ATR-SEIRAS is acceptable compared to literature^47,48^.

To understand the effects of Ba-anchored sulfate and W on acidic OER activity, DFT calculations were performed based on the aforesaid atomic structures of RuO_2_, Ba_0.4_(SO_4_)_*δ*_Ru_0.6_O_2−*δ*_, and Ba_0.3_(SO_4_)_*δ*_W_0.2_Ru_0.5_O_2−*δ*_. Since OER takes place on the catalyst surface, our analysis followed the adsorbate-evolving mechanism (AEM), which has been widely accepted for acidic OER on RuO_2_^39,40^. Briefly, the first adsorbed water molecule undergoes two deprotonation steps on the under-coordinated Ru (Ru_cus_) site to sequentially form *OH and *O, which then adsorbs the second water molecule and deprotonates into *OOH (Fig. 4a and Supplementary Fig. 20 for detailed description). The *OOH can readily donate a proton to an adjacent O site, forming a strong hydrogen bond (denoted as *OO–H). Finally, the *OO–H intermediate is deprotonated and an O_2_ molecule is released, which is considered the RDS as reported by Shao-Horn et al.^38,41^. Weakening the too-strong adsorption energy of oxygenated intermediates on Ru_cus_ can effectively accelerate the OER process^42,43^.

As depicted by the Gibbs free energy diagram of the acidic OER process, ΔG_*OOH_ on Ba_0.4_(SO_4_)_*δ*_Ru_0.6_O_2−*δ*_ and Ba_0.3_(SO_4_)_*δ*_W_0.2_Ru_0.5_O_2−*δ*_ was 3.16 and 3.05 eV, respectively, lower than that of RuO_2_ (3.69 eV) (Fig. 4a, c, e). The O atom adjacent to *OOH—the proton acceptor—was bound to S in sulfate-containing catalysts, whereas it was bound to Ru in RuO_2_ (Supplementary Fig. 20). S is more electronegative than Ru, resulting in a lower Gibbs free energy difference (ΔG_*OOH_ − ΔG_*OO-H_) on Ba_0.4_(SO_4_)_*δ*_Ru_0.6_O_2−*δ*_ (0.05 eV) and Ba_0.3_(SO_4_)_*δ*_W_0.2_Ru_0.5_O_2−*δ*_ (0.01 eV) than that on RuO_2_ (0.81 eV)^44,45^. This further promoted the deprotonation of *OO–H—the absolute values of ($ΔG_{{{\rm{O}}}_{2}}$ − ΔG_*OO-H_) on Ba_0.4_(SO_4_)_*δ*_Ru_0.6_O_2−*δ*_ (1.27 eV) and Ba_0.3_(SO_4_)_*δ*_W_0.2_Ru_0.5_O_2−*δ*_ (1.32 eV) were lower than on RuO_2_ (1.5 eV). These results clearly attributed the accelerated OER kinetics to the addition of sulfate, which weakened the adsorption energy of oxygenated intermediates. The DFT calculations were validated by in-situ attenuated total reflection surface-enhanced infrared absorption spectroscopy (ATR-SEIRAS). When applying oxidation potentials from 1.1 to 1.8 V on RuO_2_, (SO_4_)_*δ*_RuO_2−*δ*_, and Ba_0.3_(SO_4_)_*δ*_W_0.2_Ru_0.5_O_2−*δ*_, a peak emerged between 1120 and 1020 cm^−1^, arising from the *OO stretching vibration in *OOH or *OO–H^46,47^. The peak intensity increased as the potential became more positive. The *OO vibration was blue-shifted from 1118 cm^−1^ on RuO_2_ to 1056 and 1050 cm^−1^ on (SO_4_)_*δ*_RuO_2−*δ*_ and Ba_0.3_(SO_4_)_*δ*_W_0.2_Ru_0.5_O_2−*δ*_, respectively (Fig. 4b, d, f). This indicated that sulfate weakened the adsorption energy of the oxygenated intermediates, in consistency with the DFT predictions. The broad peak between 1250 and 1150 cm^−1^ was assigned to the Si-O-Si vibration from the ATR crystal or the S = O vibrations of sulfate^48,49^.

The weakened adsorption energy of oxygenated intermediates in the presence of sulfate accelerated the dehydrogenation of *OO–H. Consequently, the RDS may switch from the dehydrogenation of *OO–H to the formation of *OOH. This hypothesis was evidenced by changes in Tafel plots^43^. As shown in Fig. 3b, the Tafel slope decreased from 83.8 mV dec^−1^ on RuO_2_ to ~44 mV dec^−1^ when sulfate was incorporated. The changed Tafel slope was close to the theoretical value of 39 mV dec^−1^ when *OOH formation is the RDS on the RuO_2_ (110) plane, as calculated by Nørskov et al.^50^, implying the formation of *OOH to be the RDS on sulfate-containing catalysts. On the other hand, the absolute value of (ΔG_*O_ − ΔG_*OOH_)—the descriptor for the energy barrier of deprotonating the second adsorbed water molecule to form *OOH—was lower on Ba_0.3_(SO_4_)_*δ*_W_0.2_Ru_0.5_O_2−*δ*_ (1.57 eV) than that on Ba_0.4_(SO_4_)_*δ*_Ru_0.6_O_2−*δ*_ (1.67 eV), indicating that the W dopant promotes the formation of *OOH (Fig. 4c, e). This was experimentally validated by CV, where the redox peaks can reflect the proton-coupled electron transfer. As shown in Supplementary Fig. 21, the oxidation peak near 1.3 V on commercial RuO_2_ corresponded to the formation of *OOH via the deprotonation of the second adsorbed water molecule^43^. When incorporating Ba-anchored sulfate, the peak position was negatively shifted to ~1.23 V on Ba_0.4_(SO_4_)_*δ*_Ru_0.6_O_2−*δ*_, and it was further shifted to ~1.17 V when introducing W to form Ba_0.3_(SO_4_)_*δ*_W_0.2_Ru_0.5_O_2−*δ*_. These results suggested that the sulfate anchored by Ba changed the RDS from *OO–H dehydrogenation to *OOH formation, and the addition of W accelerated the altered RDS to collectively promote the OER activity.

### Understanding the enhanced stability on Ba_0.3_(SO_4_)_*δ*_W_0.2_Ru_0.5_O_2−*δ*_

**Fig. 5:**
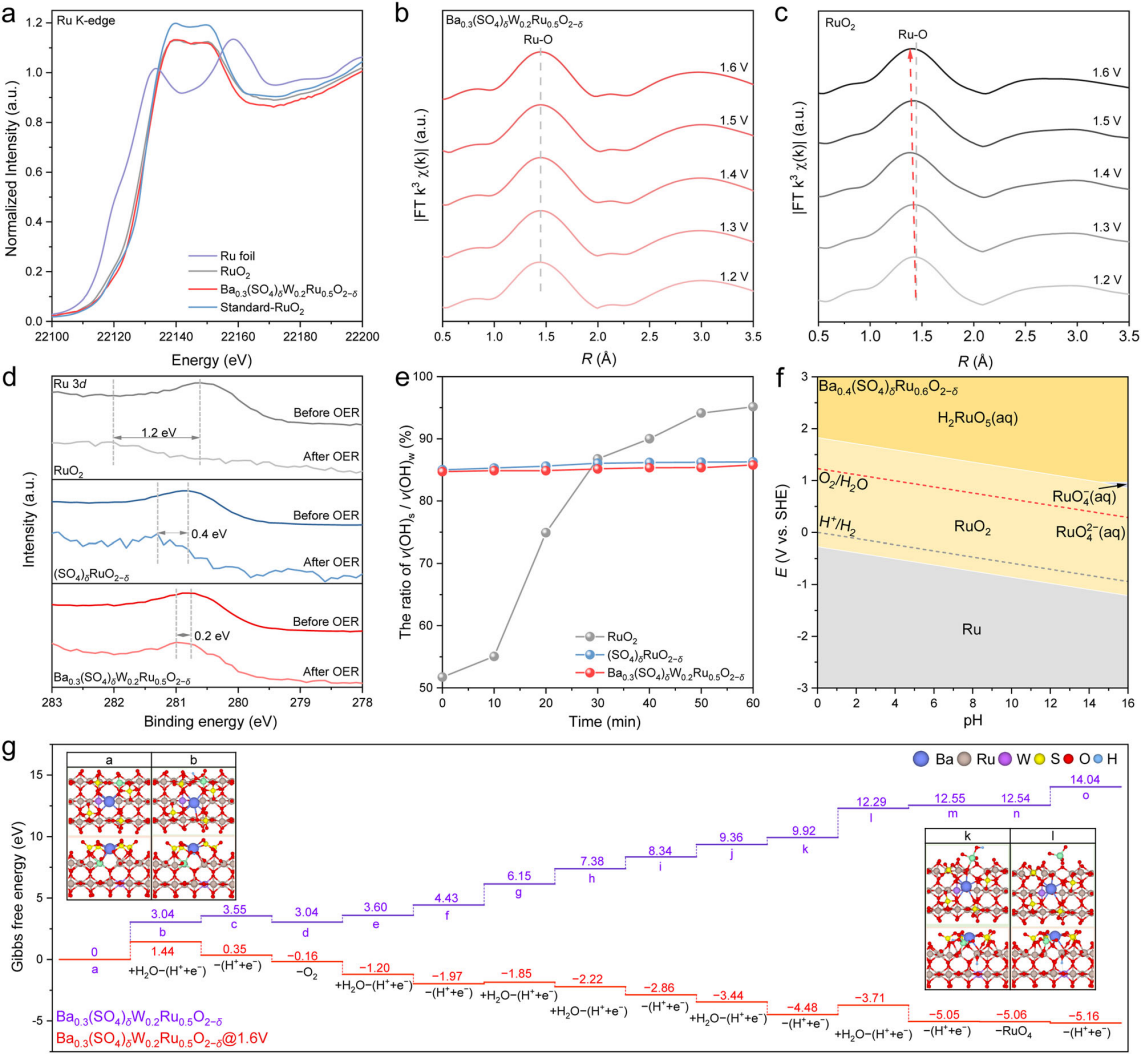
Understanding the enhanced stability on Ba_0.3_(SO_4_)_*δ*_W_0.2_Ru_0.5_O_2−*δ*_. **a** Normalized Ru K-edge XANES spectra of Ba_0.3_(SO_4_)_*δ*_W_0.2_Ru_0.5_O_2−*δ*_, RuO_2_, Ru foil, and standard-RuO_2_. **b**, **c** *Operando* EXAFS spectra of Ru edge on **b** Ba_0.3_(SO_4_)_*δ*_W_0.2_Ru_0.5_O_2−*δ*_ and **c** RuO_2_ at acidic OER potentials from 1.2 to 1.6 V. **d** High-resolution XPS spectra of Ru 3*d* for RuO_2_, (SO_4_)_*δ*_RuO_2−*δ*_, and Ba_0.3_(SO_4_)_*δ*_W_0.2_Ru_0.5_O_2−*δ*_ before and after OER. **e** The intensity ratio of *v*(OH)_s_ / *v*(OH)_w_ on RuO_2_, (SO_4_)_*δ*_RuO_2−*δ*_, and Ba_0.3_(SO_4_)_*δ*_W_0.2_Ru_0.5_O_2−*δ*_ over the course of 1 h acidic OER at 1.5 V. **f** Ru Pourbaix diagram for Ba_0.4_(SO_4_)_*δ*_Ru_0.6_O_2−*δ*_ generated with an aqueous ion concentration of 10^−6^ M at 25 °C. **g** Gibbs free energy diagram of Ru dissolution on the grain edges of Ba_0.3_(SO_4_)_*δ*_W_0.2_Ru_0.5_O_2−*δ*_.

Besides the activity, gaining insights into the noticeable stability of Ba_0.3_(SO_4_)_*δ*_W_0.2_Ru_0.5_O_2−*δ*_ may be even more important for practical applications. The deactivation of RuO_2_ originates from the formation of V_o_ due to the overoxidation of lattice O at high-oxidation potentials. Consequently, the adjacent surface Ru atoms are oxidized into soluble high-valence Ru^x>4^ derivatives^17^. With this notion, we performed X-ray absorption spectroscopy (XAS) on Ba_0.3_(SO_4_)_*δ*_W_0.2_Ru_0.5_O_2−*δ*_, RuO_2_, Ru foil, and standard-RuO_2_. The Ru K-edge X-ray absorption near edge structure (XANES) was shown in Fig. 5a. Benchmarked by the standard-RuO_2_, the Ru oxidation state in Ba_0.3_(SO_4_)_*δ*_W_0.2_Ru_0.5_O_2−*δ*_ was very close to +4, slightly higher than that in RuO_2_. Ru-based materials with a higher valence state of Ru possess higher OER activity according to previous studies, consistent with our results^38,51^. *Operando* k^3^*-*weighted Fourier transformed extended X-ray absorption fine structure (FT-EXAFS) spectra were obtained to analyze the structural changes of catalysts during acidic OER, and the fitting parameters and results are listed in Supplementary Table 6 and Supplementary Fig. 22. For Ba_0.3_(SO_4_)_*δ*_W_0.2_Ru_0.5_O_2−*δ*_, the scattering peak at 1.97 Å was ascribed to Ru–O coordination on the first shell of the nanoparticles. The peak remained unchanged over the OER potentials from 1.2 to 1.6 V (Fig. 5b), indicating the stable structure of Ba_0.3_(SO_4_)_*δ*_W_0.2_Ru_0.5_O_2−*δ*_ under acidic OER conditions. In contrast, the peak of Ru–O coordination for RuO_2_ was negatively shifted as the potential increased (Fig. 5c). This implied that the formation of V_o_ and the dissolution of Ru occurred at high-oxidation potentials, thereby lowering the Ru–O coordination number and shortening the interatomic distance^52^.

Figure 5d shows the Ru 3*d* XPS spectra of RuO_2_, (SO_4_)_*δ*_RuO_2−*δ*_, and Ba_0.3_(SO_4_)_*δ*_W_0.2_Ru_0.5_O_2−*δ*_ before and after OER, and the peak at ~280.7 eV was assigned to Ru^4+^ species^53^. After OER, the binding energy of Ru 3*d* on the tested catalysts shifted positively in the following order: RuO_2_ (1.2 eV) > (SO_4_)_*δ*_RuO_2−*δ*_ (0.4 eV) ≈ Ba_0.3_(SO_4_)_*δ*_W_0.2_Ru_0.5_O_2−*δ*_ (0.2 eV). This implied that the addition of sulfate effectively impeded the oxidation of Ru^4+^ species to Ru^x>4^ derivatives during acidic OER. The protection of RuO_2_ by sulfate was confirmed by the O 1 *s* XPS spectra, which were deconvoluted into three peaks at 529.8, 531.9, and 533.2 eV corresponding to M-O bonds (O^2−^), hydroxyl groups (OH^−^), and adsorbed water (H_2_O), respectively^54^. The Ru–O and Ru-OH bonds were observed on all catalysts before OER, arising from the intrinsic properties of oxides (Supplementary Fig. 23)^55^. After the OER test, the Ru–O peak of RuO_2_ vanished, whereas that of (SO_4_)_*δ*_RuO_2−*δ*_ and Ba_0.3_(SO_4_)_*δ*_W_0.2_Ru_0.5_O_2−*δ*_ was attenuated but remained obvious. This indicated that sulfate can stabilize the lattice O during acidic OER. The structural changes of the catalysts were further investigated using time-resolved in-situ XRD at a high-oxidation potential of 1.55 V. The XRD patterns of RuO_2_ indicated a gradual weakening (~34.7% decrease) of the characteristic (101) peak density at 2θ = 35.1° (JCPDS 88-0286) during the continuous 200 min acidic OER due to the dissolution of nanoparticles (Supplementary Fig. 24), consistent with the XPS results. On the contrary, the characteristic peak intensity of Ba_0.3_(SO_4_)_*δ*_W_0.2_Ru_0.5_O_2−*δ*_ reduced negligibly throughout the reaction (Supplementary Fig. 25), suggesting its stable structure under acidic OER conditions.

As predicted by theory, sulfate could inhibit the formation of V_o_ by forming coordination-saturated lattice O via bonding its under-coordinated O with under-coordinated Ru. To verify this hypothesis, we performed time-resolved in-situ ATR-SEIRAS on RuO_2_, (SO_4_)_*δ*_RuO_2−*δ*_, and Ba_0.3_(SO_4_)_*δ*_W_0.2_Ru_0.5_O_2−*δ*_ at 1.5 V. The O–H stretching modes of the interfacial H-bonded water network were deconvoluted into three Gaussian peaks^49,56,57^: (1) isolated non-H-bonded water at ~3600 cm^−1^ (i.e., free water, with free O–H bonds denoted as *v*(OH)_i_), (2) trihedral H-bonded water at ~3400 cm^−1^ (i.e., liquid-like water, with relatively weak O–H bonds denoted as *v*(OH)_w_), and (3) tetrahedral H-bonded water at ~ 3200 cm^−1^ (i.e., ice-like water, with strong O–H bonds denoted as *v*(OH)_s_). According to experimental and theoretical studies on aqueous-solid interfacial interactions, *v*(OH)_s_ is bound to 1f-cus Ru sites and *v*(OH)_w_ is bound to lattice O via H bonds^58,59^. As shown in the time-resolved in-situ ATR-SEIRAS spectra, *v*(OH)_i_, *v*(OH)_w_, and *v*(OH)_s_ were all detected on Ru-based catalysts during OER (Supplementary Fig. 26). On RuO_2_, the proportion of *v*(OH)_w_ gradually decreased and the intensity ratio of *v*(OH)_s_/*v*(OH)_w_ increased with time (Fig. 5e), which can be attributed to the formation of V_o_ due to the over-oxidation of lattice O. In contrast, on (SO_4_)_*δ*_RuO_2−*δ*_ and Ba_0.3_(SO_4_)_*δ*_W_0.2_Ru_0.5_O_2−*δ*_, *v*(OH)_w_ remained dominant and the intensity ratio of *v*(OH)_s_/*v*(OH)_w_ was almost unchanged during acidic OER, entailing that sulfate enhanced the protection of lattice O and impeded the formation of V_o_.

The functions of Ba-anchored sulfate in protecting lattice O motivated us to carry out DFT studies to better understand its stability mechanism for RuO_2_. Pourbaix analysis was conducted to assess the phase stability of RuO_2_ and Ba_0.4_(SO_4_)_*δ*_Ru_0.6_O_2−*δ*_ (Supplementary Table 7), which can be quantitatively evaluated by computing the Pourbaix decomposition free energy (ΔG_pbx_) of the catalysts with respect to the stable domains in the Pourbaix diagram as a function of pH and potential^8,60^. Supplementary Fig. 27 shows a typical Pourbaix diagram for the Ru element in aqueous solutions. The upper and lower dashed lines indicate the thermodynamic potentials for OER and hydrogen evolution reaction (HER) at different pH values, respectively^61^. In an acidic environment (pH <7), RuO_2_ is stable within a narrow potential range, and it transformed into soluble H_2_RuO_5_ at 1.32 V when pH = 0. In contrast, the RuO_2_ phase of Ba_0.4_(SO_4_)_*δ*_Ru_0.6_O_2−*δ*_ remained stable over a wider OER potential range, and it was intact until the potential increased to 1.83 V at pH = 0 (Fig. 5f). In addition, the ΔG_pbx_ of Ba_0.4_(SO_4_)_*δ*_Ru_0.6_O_2−*δ*_ is 0.69 eV atom^−1^ at 2 V when pH = 0, lower than that of the RuO_2_ (2.74 eV atom^−1^) (Supplementary Fig. 28)—this confirmed the stability of Ba_0.4_(SO_4_)_*δ*_Ru_0.6_O_2−*δ*_^8^. Furthermore, we also evaluated the thermodynamic stability of the structure of Ba-anchored sulfate. The calculation indicates that Ba-anchored sulfate is highly stable when pH <13.6 (Supplementary Fig. 29), consistent with the ICP-MS result (Supplementary Table 4). Moreover, the Ru dissolution process on the grain edges of Ba_0.3_(SO_4_)_*δ*_W_0.2_Ru_0.5_O_2−*δ*_ was further investigated during prolonged acidic OER (Supplementary Fig. 30). As depicted in Fig. 5g, with the coordination-saturated O shared by sulfate and Ru as the adsorption site at 1.6 V, the Gibbs free energy difference of water adsorption and deprotonation (*a* to *b*) on Ba_0.3_(SO_4_)_*δ*_W_0.2_Ru_0.5_O_2−*δ*_ was 1.44 eV, significantly higher than that on RuO_2_ (−0.05 eV, Fig. 1a). This indicated that the shared O was difficult to be over-oxidized, consistent with the in-situ ATR-SEIRAS results. In subsequent steps, the dissolution of Ru was almost spontaneous on RuO_2_, whereas on Ba_0.3_(SO_4_)_*δ*_W_0.2_Ru_0.5_O_2−*δ*_, the dehydrogenation of *RuO_4_-H (*k* to *l*) needed to overcome an energy barrier of 0.77 eV. These results collectively ascertained that incorporating Ba-anchored sulfate can effectively inhibit the over-oxidation of shared O, hinder the Ru loss, and stabilize the RuO_2_ crystal structure.

### PEMWE performance in 0.5 M H_2_SO_4_

**Fig. 6:**
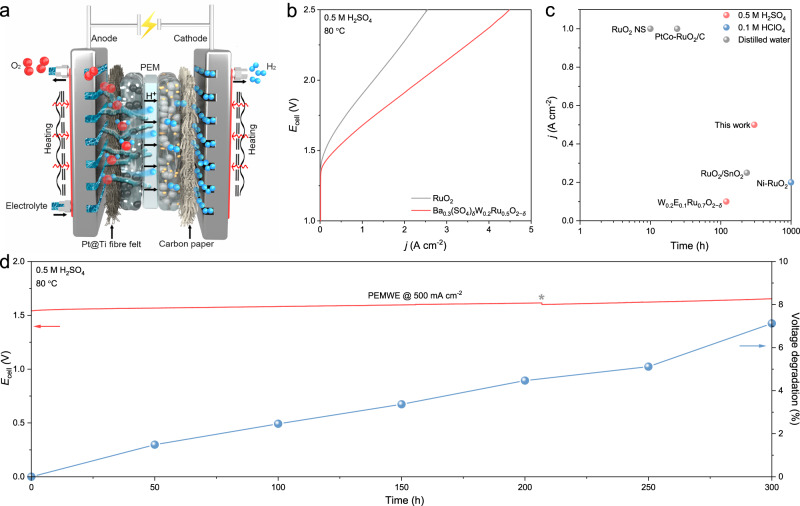
Stability of Ba_0.3_(SO_4_)_*δ*_W_0.2_Ru_0.5_O_2−*δ*_ in PEMWE. **a** Schematic of the PEMWE. **b** Polarization curves of the PEMWEs using commercial RuO_2_ and Ba_0.3_(SO_4_)_*δ*_W_0.2_Ru_0.5_O_2−*δ*_ as the anode catalysts in 0.5 M H_2_SO_4_ at 80 °C. **c** PEMWE stability based on Ba_0.3_(SO_4_)_*δ*_W_0.2_Ru_0.5_O_2−*δ*_ and other reported Ru-based anode catalysts (Supplementary Table 8). **d** Chronopotentiogram and voltage degradation of Ba_0.3_(SO_4_)_*δ*_W_0.2_Ru_0.5_O_2−*δ*_-based PEMWE operated at 500 mA cm^−2^ in 0.5 M H_2_SO_4_ under 80 °C. Of note, fresh electrolyte was replenished at the 206th h (gray asterisk).

Last, we sought to operate the Ba_0.3_(SO_4_)_*δ*_W_0.2_Ru_0.5_O_2−*δ*_ catalyst in practical applications. A membrane electrode assembly (MEA) based PEMWE (Supplementary Fig. 31) was constructed with Ba_0.3_(SO_4_)_*δ*_W_0.2_Ru_0.5_O_2−*δ*_ as the anode catalyst, commercial Pt/C as the cathode catalyst, and Nafion 115 as the proton-exchange membrane (Fig. 6a). As the benchmark, a similar PEMWE was fabricated using commercial RuO_2_ as the anode catalyst. All device performances were evaluated in 0.5 M H_2_SO_4_ at 80 °C without iR correction. As compared in Fig. 6b, to reach water-splitting current densities of 0.5, 1, and 2 A cm^−2^, the Ba_0.3_(SO_4_)_*δ*_W_0.2_Ru_0.5_O_2−*δ*_-based PEMWE required full-cell voltages of 1.54, 1.68, and 1.91 V, respectively, much lower than those of the RuO_2_-based PEMWE (1.70, 1.90, and 2.28 V). Remarkably, further increasing the full-cell voltage to 2.5 V facilitated the Ba_0.3_(SO_4_)_*δ*_W_0.2_Ru_0.5_O_2−*δ*_-based PEMWE to operate at a current density of 4.49 A cm^−2^, corresponding to a hydrogen production rate of ~1.88 L_H2_ h^−1^. The Ba_0.3_(SO_4_)_*δ*_W_0.2_Ru_0.5_O_2−*δ*_-based PEMWE also incurred a low electricity consumption of ~42.9 kW h kg^−1^_H2_ and a power density of 1.17 W cm^−2^ at 1.6 V, which were aligned with existing PEMWEs in the market (electricity consumptions between 47 and 73 kW h kg^−1^_H2_ and power densities between 1.40 and 3.52 W cm^−2^ at 1.6 V)^62^.

More impressively, the Ba_0.3_(SO_4_)_*δ*_W_0.2_Ru_0.5_O_2−*δ*_-based PEMWE demonstrated markedly improved device stability at 500 mA cm^−2^ in 0.5 M H_2_SO_4_. As shown in Fig. 6d, the Ba_0.3_(SO_4_)_*δ*_W_0.2_Ru_0.5_O_2−*δ*_-based PEMWE can be operated stably for 300 h and the voltage increased by only ~7.1%. In contrast, the voltage of the RuO_2_-based PEMWE increased by 400 mV in just a few hours (Supplementary Fig. 32). Furthermore, we also conducted a test on the Ba_0.3_(SO_4_)_*δ*_W_0.2_Ru_0.5_O_2−*δ*_-based PEMWE at 1 A cm^−2^ in distilled water (Supplementary Fig. 33). After 300 h stability test, the voltage increased by only ~4.9%, indicating its excellent stability. Figure 6c and Supplementary Table 8 summarize the active lifetime of Ba_0.3_(SO_4_)_*δ*_W_0.2_Ru_0.5_O_2−*δ*_ and other state-of-the-art Ru-based OER catalysts in PEMWEs as a function of current density in distilled water, 0.1 M HClO_4_, and 0.5 M H_2_SO_4_. Although operated in strong acid at a high current density, our Ba_0.3_(SO_4_)_*δ*_W_0.2_Ru_0.5_O_2−*δ*_ catalyst manifested exceptional device stability metrics.

## Discussion

In summary, toward the desired goal of stabilizing RuO_2_ for acidic OER, we not only elucidated the catalyst deactivation mechanism, but also shed light on how theory can guide material design principles. Based on our oxyanion protection strategy, the designed Ba-anchored sulfate effectively prevented the loss of surface Ru atoms by inhibiting the overoxidation of lattice O, and W dopant lowered the over-potential to further enhance the acidic OER stability. The resulting Ba_0.3_(SO_4_)_*δ*_W_0.2_Ru_0.5_O_2−*δ*_ catalyst, when operated in a PEMWE, delivered a 300 h stability at 500 mA cm^−2^ in 0.5 M H_2_SO_4_, verifying the effectiveness of the strategy. In a broader context, this work can be translated to acidic water electrolysis at a larger scale, underpinned by further improvements in stability and a decrease in operating voltage.

## Methods

### Catalyst synthesis

All chemicals and reagents were used as received without further purification. All aqueous solutions were prepared with distilled water (Millipore, 18.2 MΩ cm). A typical Schlenk line was used to synthesize the Ba_m_(SO_4_)_*δ*_W_n_Ru_1−m−n_O_2−*δ*_ nanoparticles. For Ba_0.3_(SO_4_)_*δ*_W_0.2_Ru_0.5_O_2−*δ*_, 0.1 mmol of ruthenium(III) acetylacetonate (Aladdin, 99.95%), 0.02 mmol of barium tungstate (Aladdin, 99.9%), and 0.6 mmol of thiourea (Aladdin, 99.0%) were dissolved in 10 mL oleylamine (Aladdin, 80–90%) by ultrasonication for 30 min. The solution was then stirred for another 30 min under an N_2_ flow. Next, the solution was heated to 250 °C for 2.5 h under N_2_ flow conditions, and cooled to room temperature after the reaction. The nanoparticles were collected by centrifugation at 12.3 × *g* for 4 min and washed at least five times with absolute ethanol (Aladdin, 97.0%). After that, the nanoparticles were redispersed in 50 mL cyclohexane (Aladdin, 99.0%) with 23 mg of carbon powder (Vulcan XC-72), then stirred and ultrasonicated for 10 h. The resulting mixture was suction-filtered and dried at 80 °C for 5 h. The product was calcined at 400 °C for 5 h using a tube oven under air conditions, followed by further acid-washing to remove impurities in 0.5 M H_2_SO_4_ for 12 h. Finally, the Ba_0.3_(SO_4_)_*δ*_W_0.2_Ru_0.5_O_2−*δ*_ catalyst was obtained after suction filtering. Of note, the physical characterizations and PEMWE tests of the Ba_0.3_(SO_4_)_*δ*_W_0.2_Ru_0.5_O_2−*δ*_ nanoparticles were conducted without carbon support. The preparation methods for as-prepared RuO_2_, W_0.3_Ru_0.7_O_2_, Ba_0.4_Ru_0.6_O_2_, (SO_4_)_*δ*_RuO_2−*δ*_, Ba_0.4_(SO_4_)_*δ*_Ru_0.6_O_2−*δ*_, and W_0.3_(SO_4_)_*δ*_Ru_0.7_O_2−*δ*_ were similar to that of Ba_0.3_(SO_4_)_*δ*_W_0.2_Ru_0.5_O_2−*δ*_, except that 0.02 mmol of barium (II) acetylacetonate (Aladdin, 99.0%) or 0.02 mmol of tungsten hexacarbonyl (Aladdin, 99.9%) were used as Ba or W precursors, and the reaction temperature for Ba_0.4_Ru_0.6_O_2_ and Ba_0.4_(SO_4_)_*δ*_Ru_0.6_O_2−*δ*_ was 300 °C.

### Theory calculation method

DFT calculations were performed by the Vienna Ab-initio Simulation Package (VASP.5.4.4) code^63^. The core-valence interactions were calculated using projector-augmented wave (PAW) pseudopotentials with a cut-off energy of 500 eV^64^. The exchange-correlation correction effect was treated by the generalized gradient approximation–Perdew–Burke–Ernzerhof (GGA-PBE) method^65^. A 20 Ångström-thick vacuum layer was used to eliminate the interaction between two adjacent slabs. The convergence accuracy was considered to be reached when the force of each atom was less than 0.02 eV·Å^−1^. The Brillouin zone was sampled on the Gamma-centered Monkhorst-Pack (MP) grids^66^, and the K-points were set as 3 × 3 × 1 in all models. The DFT-D3(BJ) method was used to consider the dispersion energy correction of van der Waals force^67^. The RuO_2_ (110) facet was constructed to simulate the OER process. In the Ba_0.3_(SO_4_)_*δ*_W_0.2_Ru_0.5_O_2−*δ*_ model, both Ba and W atoms were uniformly doped into the lattice sites of Ru with a metal atomic concentration of around 8.5%. Four enriched SO_4_ groups (i.e., sulfate groups) were uniformly anchored by one surface Ba atom. In each sulfate, two O atoms replaced two surface lattice O of RuO_2_ to bond with Ru, and one O atom was bound with Ba. To screen the M in the periodic table, the sulfate-enriched surface Ba was replaced by the other elements. In the Ru loss model, the boundary was constructed by two perpendicular (110) and (−110) facets. Data processing was assisted by VASPKIT^68^, QVASP^69^, and VESTA^70^ software. In particular, the exact energy of the triplet O_2_ (–10.33 eV) was determined by VAPKIT. Porbaix diagrams were calculated using Atomic Simulation Environment (ASE) with input formation energy by DFT calculations of bulk and surface models^71^. The Gibbs free energy difference (ΔG) between the initial and final states was defined by ΔG = ΔE + ΔZPE − TΔS, where E, ZPE, T, and S represent the DFT-calculated energy, zero-point energy, temperature (298.15 K) and entropy, respectively^72^.

### Characterization

TEM images were acquired using an FEI Tecnai G2 Spirit Twin transmission electron microscope operating at 120 kV. HAADF–STEM, SAED, and EDS elemental mappings were performed by an FEI Titan 80-300 equipped with a field emission gun and spherical aberration corrector working at an accelerating voltage of 300 kV. XRD patterns of each sample were collected on a Bruker D8 Advance with Cu K_α_ radiation (λ = 0.154 nm). The data were collected with a step size of 0.01 s and a dwell time of 0.1 s. XPS spectra were recorded at a Kratos Analytical AMICUS/ESCA 3400 equipped with an Mg-anode K_α_ excitation X-ray source (*hν* = 1253.6 eV) at 10 kV, 10 mA, and 2 × 10^−6^ Pa. All binding energies of the elements were referred to the C 1 s peak at 284.8 eV. XPS spectra of catalysts after the OER were obtained when the CV curves became stable. ICP-MS (Agilent, 8800) was used to determine the contents of the elements for as-prepared catalysts. Prior to ICP-MS measurements, the samples were digested by an Ultrawave (Milestone, SRC Technology) in aqua regia at 250 °C under 50 bar for 12 h. The S-number of catalysts was calculated according to the definition by Geiger et al.^73^: S-number = ${n}_{{O}_{2}}/{n}_{{Ru}}$. The moles of the evolved oxygen $({n}_{{O}_{2}})$ which can be determined by integrating the current (*i*) during the constant current: ${n}_{{O}_{2}}=1/{zF}\int i(t){dt}$, where z is the number of electrons transferred during the reaction (z = 4 for OER), F is the Faraday constant (96485 C mol^−1^), and *t* is the reaction time. The moles of the dissolved Ru (${n}_{{Ru}}$) can be determined by: ${n}_{{Ru}}={m}_{{Ru}}/{M}_{{Ru}}$, where ${m}_{{Ru}}$ is the mass of dissolved Ru during OER, ${M}_{{Ru}}$ is the molar mass of Ru (101.07 g mol^−1^).

*Operando* XAS measurements were performed using the beamline BL01C1 at the National Synchrotron Radiation Research Center (NSRRC, Taiwan). First, the catalyst was dropped onto the carbon paper as the working electrode with a catalyst loading of 0.1 mg cm^−2^. A carbon bar and an Ag/AgCl electrode were used as the counter and reference electrodes, respectively. The working electrode was assembled into an organic glass electrochemical cell with 0.5 M H_2_SO_4_ as the electrolyte. The spectra were collected at oxidation potentials of 1.2, 1.3, 1.4, 1.5, and 1.6 V. Ru foil and standard-RuO_2_ were tested as the references. Data processing and EXAFS fitting were carried out using the Athena and Artemis programs^74^.

In-situ XRD was carried out on a Bruker D8 Discover XRD spectrometer equipped with an IμS microfocus X-ray radiation source and an Eiger 2D detector. A collimator (2 mm in diameter) was used to improve the X-ray intensity of the laser spot. A 2D detector was vertically positioned to collect the information in the still scan mode. An optical laser-video camera system was integrated for accurate sample positioning and system calibration. The catalyst was sprayed on carbon paper as the working electrode, which was positioned on the outermost layer of a customized electrolytic cell and wrapped with adhesive tape (without any XRD diffraction signal)^75^. A Pt wire and an Ag/AgCl electrode were used as the counter and reference electrodes, respectively. In total, 0.5 M H_2_SO_4_ was used as the electrolyte. A constant potential of 1.55 V (without iR correction) was applied to the working electrode by an electrochemistry workstation (VSP-3e, Bio-Logic). In-situ XRD patterns were acquired in the still scan mode with an integration time of 20 min.

In-situ ATR-SEIRAS were performed using a Nicolet iS50 FT-IR spectrometer equipped with an MCT detector cooled with liquid nitrogen and a PIKE VeeMAX III variable angle ATR sampling accessory. The spectral resolution was 4 cm^−1^, and 64 interferograms were co-added for each spectrum. The spectra are shown in absorption units defined as A = −log(R/R_0_), where R and R_0_ represent the reflected IR intensities of the samples and the reference single beam spectrum, respectively. The ATR crystal was a Si face-angled crystal with an incident angle of 60°. After polishing the crystal, an ultra-thin Au film was coated on the surface using a chemical method to enhance the IR signal and electron conduction^76^. The catalyst ink without Nafion was dropped onto the Au film as a working electrode, and 0.5 M H_2_SO_4_ solution was used as the electrolyte. A graphite rod and an Ag/AgCl electrode were used as the counter and reference electrodes, respectively. All SEIRAS spectra were collected using the CP method (1.1–1.8 V, without iR correction), and the spectrum obtained at 1.0 V was used as the reference.

### Electrochemical measurements on RDE

The OER activity of the catalysts was measured on RDE in a typical three-electrode system using 0.5 M H_2_SO_4_ as the electrolyte at 25 °C. The working electrode was a catalyst-coated glassy carbon (GC) electrode (5 mm in diameter) rotated at 1600 rpm. A Pt wire and an Ag/AgCl electrode were used as the counter and reference electrodes, respectively. The homogeneous catalyst ink was prepared by dispersing 2 mg of catalyst, 990 μL of absolute ethanol, and 10 μL of Nafion solution (Dupont, 5%) under ultrasonication in an ice-bath for 5 h. The catalyst ink was drop-casted on the GC electrode to form a thin film with a catalyst loading of 125 μg_oxide_ cm^−2^. The OER stability of the catalysts was tested on carbon paper with the same loading due to the long-term operation. All potentials were converted to RHE with iR correction. Of note, Pt/C was used as the working electrode with a rotation rate of 1600 rpm in an H_2_-saturated 0.5 M H_2_SO_4_ electrolyte to identify the equilibrium potential of hydrogen evolution/oxidation reaction (the zero potential of RHE). The OER polarization curves of catalysts were obtained from 1.1 to 1.6 V at a scan rate of 10 mV s^−1^. The accelerated aging tests were conducted via fast-scan CV from 1.2 to 1.45 V at a scan rate of 100 mV s^−1^ for 100,000 cycles. EIS was performed from 10,000 to 0.1 Hz with a voltage perturbation of 10 mV at 1.5 V. The *C*_dl_ was determined by non-Faradaic capacitive currents (*i*_c_) at different scan rates (ν) of 20, 60, 100, 140, and 180 mV s^−1^ between 1.21 and 1.32 V, and was calculated by *i*_c_ = ν*C*_dl_. ECSA was obtained from *C*_dl_ by ECSA = *C*_dl_/*C*_s_, where *C*_s_ is 0.035 mF cm^−2^ according to the reported specific capacitance^77^.

### PEMWE tests

The as-synthesized Ba_0.3_(SO_4_)_*δ*_W_0.2_Ru_0.5_O_2−*δ*_ or commercial RuO_2_ (Alfa Aesar, 99.9%) was used as the anode catalyst for PEMWE with a loading of ~3 mg_oxide_ cm^−2^. Commercial Pt/C (Alfa Aesar, 60 wt% Pt) was used as the cathode catalyst with a loading of ~0.5 mg_Pt_ cm^−2^. Nafion 115 served as the PEM. Prior to usage, the PEM was sequentially treated at 80 °C for 30 min with H_2_O_2_ (Aladdin, 5%), 0.5 M H_2_SO_4_, and distilled water. Pt-plated Ti fiber felt and carbon paper were used as anode and cathode gas diffusion layers (GDLs), respectively. The catalyst inks for both anode and cathode were prepared by ultrasonically mixing the catalyst, Nafion (Dupont, 5%), isopropanol, and distilled water at weight ratios of 1:6:80:10 and 1:10:80:10, respectively, for 1 h under ice-bath conditions (0 °C). Subsequently, half of the catalyst ink was sprayed onto the PEM and the other half was sprayed onto the GDL surfaces. The catalyst-loaded PEM and GDLs were then hot-pressed together at 120 °C for 3 min under a pressure of 0.3 MPa to fabricate the MEA. The MEA was sandwiched by two Ti bipolar plates to complete a PEMWE. Each Ti bipolar plate possessed a serpentine flow channel with a reactive area of 1 cm × 1 cm. The PEMWEs were tested at a temperature of 80 °C using 0.5 M H_2_SO_4_ or distilled water as the electrolyte, which was circulated to the anode by a peristaltic pump (LongerPump, BT100-3J) at a flow rate of 10 mL min^−1^. Polarization curves of the PEMWEs were obtained from 1.0 to 2.5 V at a scan rate of 10 mV s^−1^. The PEMWE stability was evaluated at a water-splitting current density of 500 mA cm^−2^ in 0.5 M H_2_SO_4_ or 1 A cm^−2^ in distilled water. All voltages measured in PEMWEs were obtained without iR correction. The hydrogen production rate (*Q*) was calculated by: *Q* = (*i* × *S* × *η* × *V*_*mol*_ × 3600)/2 *F*, where *i* is the current density, *S* is the reactive area of the MEA, *η* is the current efficiency (assumed as 100%), *V*_mol_ is the molar volume (22.43 L mol^−1^ at standard temperature and pressure), and *F* is the Faraday constant (96,500 C mol^−1^). The electricity consumption (*W*) was calculated from *Q* by: *W* = *P* × *n* / (*Q* × *ρ*), where *P* is the power density, *n* is the area of MEA, and *ρ* is the density of hydrogen (0.089 g L^−1^).

### Supplementary information

Supplementary Information

## Data Availability

Source data are provided with this paper.

## References

[1] Hao S (2021). Torsion strained iridium oxide for efficient acidic water oxidation in proton exchange membrane electrolyzers. Nat. Nanotechnol..

[2] Gasteiger HubertA, Just NMM (2009). a dream—or future reality?. Science.

[3] Lagadec MF, Grimaud A (2020). Water electrolysers with closed and open electrochemical systems. Nat. Mater..

[4] Shi Z (2022). Enhanced acidic water oxidation by dynamic migration of oxygen species at the Ir/Nb_2_O_5-x_ catalyst/support interfaces. Angew. Chem. Int. Ed..

[5] Wang Y, Pang Y, Xu H, Martinez A, Chen KS (2022). PEM fuel cell and electrolysis cell technologies and hydrogen infrastructure development—a review. Energy Environ. Sci..

[6] Chen FY, Wu ZY, Adler Z, Wang H (2021). Stability challenges of electrocatalytic oxygen evolution reaction: from mechanistic understanding to reactor design. Joule.

[7] She L (2021). On the durability of iridium‐based electrocatalysts toward the oxygen evolution reaction under acid environment. Adv. Funct. Mater..

[8] Hubert MA (2020). Acidic oxygen evolution reaction activity–stability relationships in Ru-based pyrochlores. ACS Catal..

[9] Bernt M (2019). Current challenges in catalyst development for PEM water electrolyzers. Chem. Ing. Tech..

[10] Higashi S, Beniya A (2023). Ultralight conductive IrO_2_ nanostructured textile enables highly efficient hydrogen and oxygen evolution reaction: Importance of catalyst layer sheet resistance. Appl. Catal. B: Environ..

[11] Tajuddin AAH (2022). Corrosion-resistant and high-entropic non-noble-metal electrodes for oxygen evolution in acidic media. Adv. Mater..

[12] Pu Z (2021). Electrocatalytic oxygen evolution reaction in acidic conditions: recent progress and perspectives. ChemSusChem.

[13] Over H (2012). Surface chemistry of ruthenium dioxide in heterogeneous catalysis and electrocatalysis: from fundamental to applied research. Chem. Rev..

[14] Millet P (2010). PEM water electrolyzers: from electrocatalysis to stack development. Int. J. Hydrog. Energy.

[15] Wang Q (2022). Long-term stability challenges and opportunities in acidic oxygen evolution electrocatalysis. Angew. Chem. Int. Ed..

[16] Qu H, He X, Wang Y, Hou S (2021). Electrocatalysis for the oxygen evolution reaction in acidic media: progress and challenges. Appl. Sci..

[17] Hao S (2020). Dopants fixation of ruthenium for boosting acidic oxygen evolution stability and activity. Nat. Commun..

[18] Qin Y (2022). RuO_2_ electronic structure and lattice strain dual engineering for enhanced acidic oxygen evolution reaction performance. Nat. Commun..

[19] Chen S (2019). Mn-doped RuO_2_ nanocrystals as highly active electrocatalysts for enhanced oxygen evolution in acidic media. ACS Catal..

[20] Yao Q (2021). A chemical etching strategy to improve and stabilize RuO_2_-based nanoassemblies for acidic oxygen evolution. Nano Energy.

[21] Wang C (2021). Advances in engineering RuO_2_ electrocatalysts towards oxygen evolution reaction. Chin. Chem. Lett..

[22] Chen Z, Duan X, Wei W, Wang S, Ni B (2020). Electrocatalysts for acidic oxygen evolution reaction: achievements and perspectives. Nano Energy.

[23] Zhang Y, Zhu X, Zhang G, Shi P, Wang A (2021). Rational catalyst design for oxygen evolution under acidic conditions: strategies toward enhanced electrocatalytic performance. J. Mater. Chem. A.

[24] Jin H (2022). Safeguarding the RuO_2_ phase against lattice oxygen oxidation during acidic water electrooxidation. Energy Environ. Sci..

[25] Wen Y (2021). Stabilizing highly active Ru sites by suppressing lattice oxygen participation in acidic water oxidation. J. Am. Chem. Soc..

[26] Yu Y (2022). High entropy stabilizing lattice oxygen participation of Ru-based oxides in acidic water oxidation. J. Mater. Chem. A.

[27] Sun H, Jung W (2021). Recent advances in doped ruthenium oxides as high-efficiency electrocatalysts for the oxygen evolution reaction. J. Mater. Chem. A.

[28] Over H (2002). Ruthenium dioxide, a fascinating material for atomic scale surface chemistry. Appl. Phys. A.

[29] Knapp M, Crihan D, Seitsonen AP, Over H (2005). Hydrogen transfer reaction on the surface of an oxide catalyst. J. Am. Chem. Soc..

[30] Li XY, Jin ZH, Zhou X, Lu K (2020). Constrained minimal-interface structures in polycrystalline copper with extremely fine grains. Science.

[31] Chen CC, Herhold AB, Johnson CS, Alivisatos AP (1997). Size dependence of structural metastability in semiconductor nanocrystals. Science.

[32] Liu Y (2018). Corrosion engineering towards efficient oxygen evolution electrodes with stable catalytic activity for over 6000 hours. Nat. Commun..

[33] Li H (2023). Boosting reactive oxygen species generation using inter-facet edge rich WO_3_ arrays for photoelectrochemical conversion. Angew. Chem. Int. Ed..

[34] Fan K (2018). Direct observation of structural evolution of metal chalcogenide in electrocatalytic water oxidation. ACS Nano.

[35] Nguyen TX, Su YH, Lin CC, Ting JM (2021). Self-reconstruction of sulfate-containing high entropy sulfide for exceptionally high-performance oxygen evolution reaction electrocatalyst. Adv. Funct. Mater..

[36] Chen H (2015). Heterogeneous fenton-like catalytic degradation of 2,4-dichlorophenoxyacetic acid in water with FeS. Chem. Eng. J..

[37] Sheng B (2019). Pivotal roles of MoS_2_ in boosting catalytic degradation of aqueous organic pollutants by Fe(II)/PMS. Chem. Eng. J..

[38] Wu ZY (2022). Non-iridium-based electrocatalyst for durable acidic oxygen evolution reaction in proton exchange membrane water electrolysis. Nat. Mater..

[39] Stoerzinger KA (2017). Orientation-dependent oxygen evolution on RuO_2_ without lattice exchange. ACS Energy Lett..

[40] Kuo DY (2018). Measurements of oxygen electroadsorption energies and oxygen evolution reaction on RuO_2_(110): a discussion of the Sabatier principle and its role in electrocatalysis. J. Am. Chem. Soc..

[41] Rao RR (2017). Towards identifying the active sites on RuO_2_(110) in catalyzing oxygen evolution. Energy Environ. Sci..

[42] Stoerzinger KA, Qiao L, Biegalski MD, Shao-Horn Y (2014). Orientation-dependent oxygen evolution activities of rutile IrO_2_ and RuO_2_. J. Phys. Chem. Lett..

[43] Rao RR (2020). Operando identification of site-dependent water oxidation activity on ruthenium dioxide single-crystal surfaces. Nat. Catal..

[44] Calligaris M (2004). Structure and bonding in metal sulfoxide complexes: an update. Coord. Chem. Rev..

[45] Moltved KA, Kepp KP (2019). The chemical bond between transition metals and oxygen: electronegativity, *d*-orbital effects, and oxophilicity as descriptors of metal-oxygen interactions. J. Phys. Chem. C..

[46] Du K (2022). Interface engineering breaks both stability and activity limits of RuO_2_ for sustainable water oxidation. Nat. Commun..

[47] Zhang Y (2018). Rate-limiting O–O bond formation pathways for water oxidation on hematite photoanode. J. Am. Chem. Soc..

[48] Nistal A (2011). Analysis of the interaction of vinyl and carbonyl silanes with carbon nanofiber surfaces. Carbon.

[49] Chen DJ, Xu B, Sun SG, Tong YJ (2012). Electroless deposition of ultrathin Au film for surface enhanced in situ spectroelectrochemisrty and reaction-driven surface reconstruction for oxygen reduction reaction. Catal. Today.

[50] Dickens CF, Kirk C, Nørskov JK (2019). Insights into the electrochemical oxygen evolution reaction with ab initio calculations and microkinetic modeling: beyond the limiting potential volcano. J. Phys. Chem. C..

[51] Zhao ZL (2020). Boosting the oxygen evolution reaction using defect-rich ultra-thin ruthenium oxide nanosheets in acidic media. Energy Environ. Sci..

[52] Shi Z (2023). Customized reaction route for ruthenium oxide towards stabilized water oxidation in high-performance PEM electrolyzers. Nat. Commun..

[53] Morgan DJ (2015). Resolving ruthenium: XPS studies of common ruthenium materials. Surf. Interface Anal..

[54] Foelske A, Barbieri O, Hahn M, Kötz R (2006). An X-ray photoelectron spectroscopy study of hydrous ruthenium oxide powders with various water contents for supercapacitors. Electrochem. solid-state lett..

[55] Haverkamp RG, Marshall AT, Cowie BCC (2011). Energy resolved XPS depth profile of (IrO_2_, RuO_2_, Sb_2_O_5_, SnO_2_) electrocatalyst powder to reveal core-shell nanoparticle structure. Surf. Interface Anal..

[56] Shen LF (2020). Interfacial structure of water as a new descriptor of the hydrogen evolution reaction. Angew. Chem. Int. Ed..

[57] Álvarez-Malmagro J, Prieto F, Rueda M, Rodes A (2014). In situ Fourier transform infrared reflection absortion spectroscopy study of adenine adsorption on gold electrodes in basic media. Electrochim. Acta.

[58] Siahrostami S, Vojvodic A (2015). Influence of adsorbed water on the oxygen evolution reaction on oxides. J. Phys. Chem. C..

[59] Arun Lobo HC (2003). Interaction of H_2_O with the RuO_2_(110) surface studied by HREELS and TDS. Surf. Sci..

[60] Wang Z, Guo X, Montoya J, Nørskov JK (2020). Predicting aqueous stability of solid with computed Pourbaix diagram using SCAN functional. NPJ Comput. Mater..

[61] Watanabe E, Rossmeisl J, Björketun ME, Ushiyama H, Yamashita K (2016). Atomic-scale analysis of the RuO_2_/water interface under electrochemical conditions. J. Phys. Chem. C..

[62] Shi Q, Zhu C, Du D, Lin Y (2019). Robust noble metal-based electrocatalysts for oxygen evolution reaction. Chem. Soc. Rev..

[63] Kresse G, Furthmüller J (1996). Efficiency of ab-initio total energy calculations for metals and semiconductors using a plane-wave basis set. Comput. Mater. Sci..

[64] Kresse G, Joubert D (1999). From ultrasoft pseudopotentials to the projector augmented-wave method. Phys. Rev. B.

[65] Perdew JP, Burke K, Ernzerhof M (1996). Generalized gradient approximation made simple. Phys. Rev. Lett..

[66] Chadi DJ, Cohen ML (1973). Special points in the Brillouin zone. Phys. Rev. B.

[67] Grimme S, Antony J, Ehrlich S, Krieg H (2010). A consistent and accurate ab initio parametrization of density functional dispersion correction (DFT-D) for the 94 elements H-Pu. J. Chem. Phys..

[68] Wang V, Xu N, Liu J-C, Tang G, Geng W-T (2021). VASPKIT: A user-friendly interface facilitating high-throughput computing and analysis using VASP code. Comput. Phys. Commun..

[69] Yi W, Tang G, Chen X, Yang B, Liu X (2020). qvasp: a flexible toolkit for VASP users in materials simulations. Comput. Phys. Commun..

[70] Momma K, Izumi F (2011). VESTA 3 for three-dimensional visualization of crystal, volumetric and morphology data. J. Appl. Crystallogr..

[71] Hjorth Larsen A (2017). The atomic simulation environment-a Python library for working with atoms. J. Phys. Condens. Matter.

[72] Nørskov JK (2004). Origin of the overpotential for oxygen reduction at a fuel-cell cathode. J. Phys. Chem. B.

[73] Geiger S (2018). The stability number as a metric for electrocatalyst stability benchmarking. Nat. Catal..

[74] Ravel B, Newville M (2005). ATHENA, ARTEMIS, HEPHAESTUS: data analysis for X-ray absorption spectroscopy using IFEFFIT. J. Synchrotron Radiat..

[75] Lei Q (2022). Structural evolution and strain generation of derived-Cu catalysts during CO_2_ electroreduction. Nat. Commun..

[76] Dunwell M (2017). The central role of bicarbonate in the electrochemical reduction of carbon dioxide on gold. J. Am. Chem. Soc..

[77] Lin Y (2019). Chromium-ruthenium oxide solid solution electrocatalyst for highly efficient oxygen evolution reaction in acidic media. Nat. Commun..

