# Supplementary material for: Stabilizing ruthenium dioxide with cation-anchored sulfate for durable oxygen evolution in proton-exchange membrane water electrolyzers
Source: Nat Commun. 2023 Dec 7;14:8093. doi: 10.1038/s41467-023-43977-7 (PMC10703920; doi:10.1038/s41467-023-43977-7)
Supplement: Supplementary file 1 — Supplementary Information [file 41467_2023_43977_MOESM1_ESM.pdf]

Supplementary Information for

Stabilizing ruthenium dioxide with cation-anchored sulfate for durable oxygen evolution in proton-exchange membrane water electrolyzers

Yanrong Xue,[1,2,7] Jiwu Zhao,[1,2,7] Liang Huang,[1,2] Ying-Rui Lu,[3] Abdul Malek,[1,2] Ge Gao,[1,2] Zhongbin Zhuang,[4] Dingsheng Wang,[5] Cafer T. Yavuz,[6] Xu Lu[1,2*]

[1]CCRC, Division of Physical Science and Engineering (PSE), King Abdullah University of Science and Technology (KAUST), Thuwal, 23955-6900, Kingdom of Saudi Arabia.

[2]KAUST Solar Center (KSC), PSE, KAUST, Thuwal, Kingdom of Saudi Arabia.

[3]National Synchrotron Radiation Research Center, Hsinchu 300, Taiwan.

[4]State Key Lab of Organic-Inorganic Composites, Beijing University of Chemical Technology, Beijing 100029, China.

[5]Department of Chemistry, Tsinghua University, Beijing 100084, China.

[6]Advanced Membranes and Porous Materials Center (AMPM), PSE, KAUST, Thuwal, Kingdom of Saudi Arabia.

[7]These authors contributed equally.

[*]Corresponding author. Email: Xu Lu (xu.lu@kaust.edu.sa).

Table of contents

Supplementary Figures 1-33	Page 3-36
Supplementary Notes 1-5	Page 3, 6, 16, 22, 33
Supplementary Tables 1-8	Page 37-44
Supplementary References 1-15	Page 45

Supplementary Figures

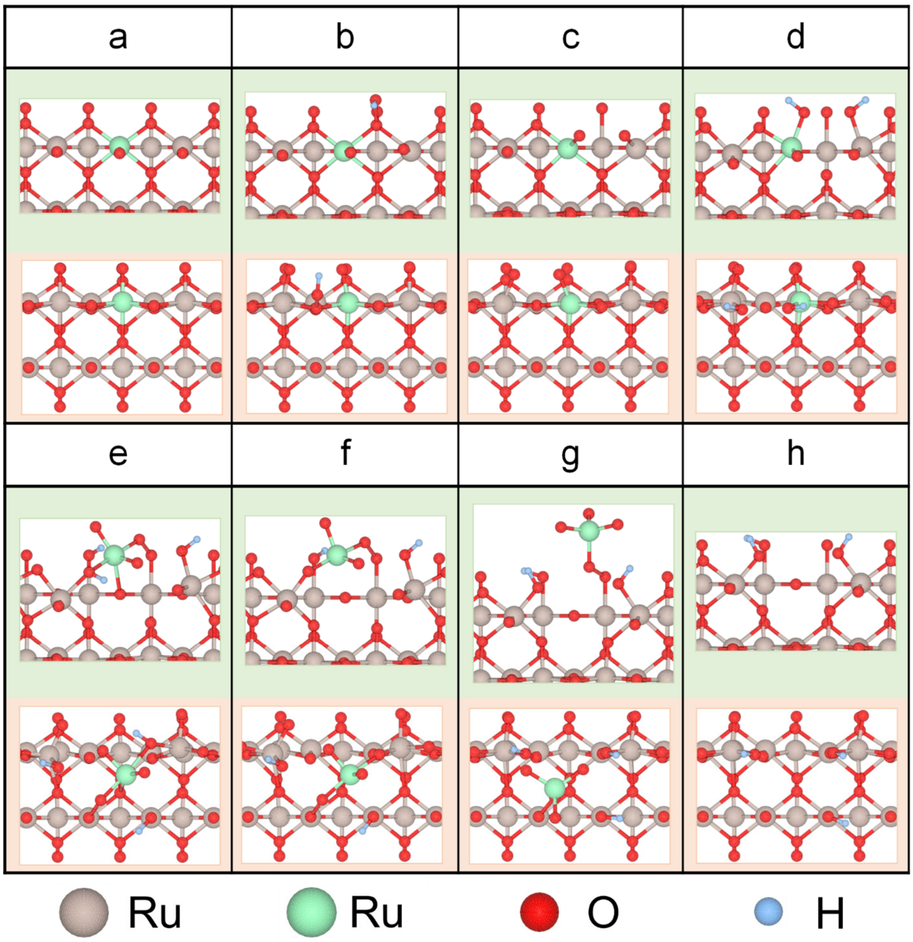

Supplementary Figure 1 Atomic structure of RuO_2 and intermediates during Ru dissolution. Of note, the green ball represents the Ru atom on the grain edge.

Supplementary Notes

Supplementary Note 1: In this work, we use the boundary between RuO_2 (110) and RuO_2 (−110) planes as our model. At first, an H_2O molecule is adsorbed onto a bridging O site on the grain edges and deprotonated to form *OH (a to b). Then, the *OH and a lattice O connected to it are deprotonated to release an O_2 molecule (b to c). In parallel, two H_2O molecules are separately adsorbed on two Ru atoms and deprotonated to form two *OH (c to d). Then, an H_2O molecule is adsorbed onto a new Ru atom adjacent to the Ru atom (green ball) and deprotonated to form *OH. At this time, the Ru-O-Ru (green ball) bond is broken, and the unstable Ru (green ball) group starts to rotate, resulting in the formation of Ru-O-O-Ru (green ball), Ru-O-Ru (the O atom from *OH), and proton transfer process (d to e). Afterward, the Ru-O-Ru (the O atom from *OH) is deprotonated to form Ru-O-Ru (e to f). Next, an H_2O molecule is adsorbed to the Ru atom and deprotonated to form *OH, accompanied by the breaking of a Ru-O-Ru bond (f to g). Finally, the RuO_4 leaves the crystal (g to h).

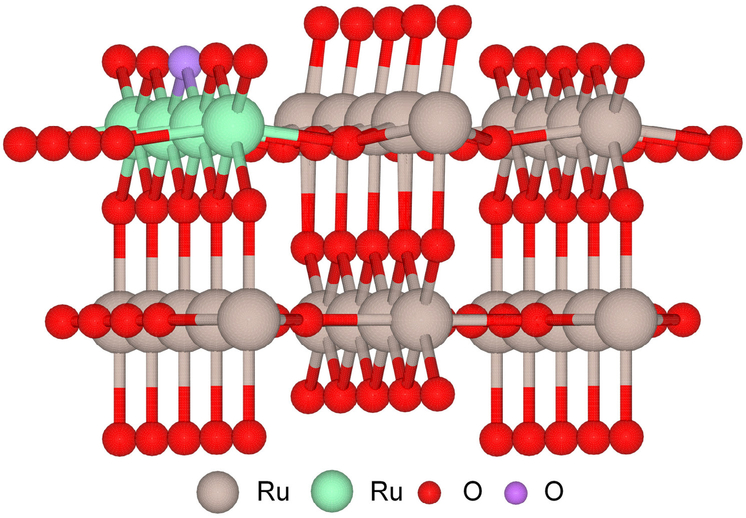

Supplementary Figure 2 Ball-and-stick model of the RuO_2 (110) plane. Of note, the green and purple balls represent Ru and O atoms, respectively, on the grain edges.

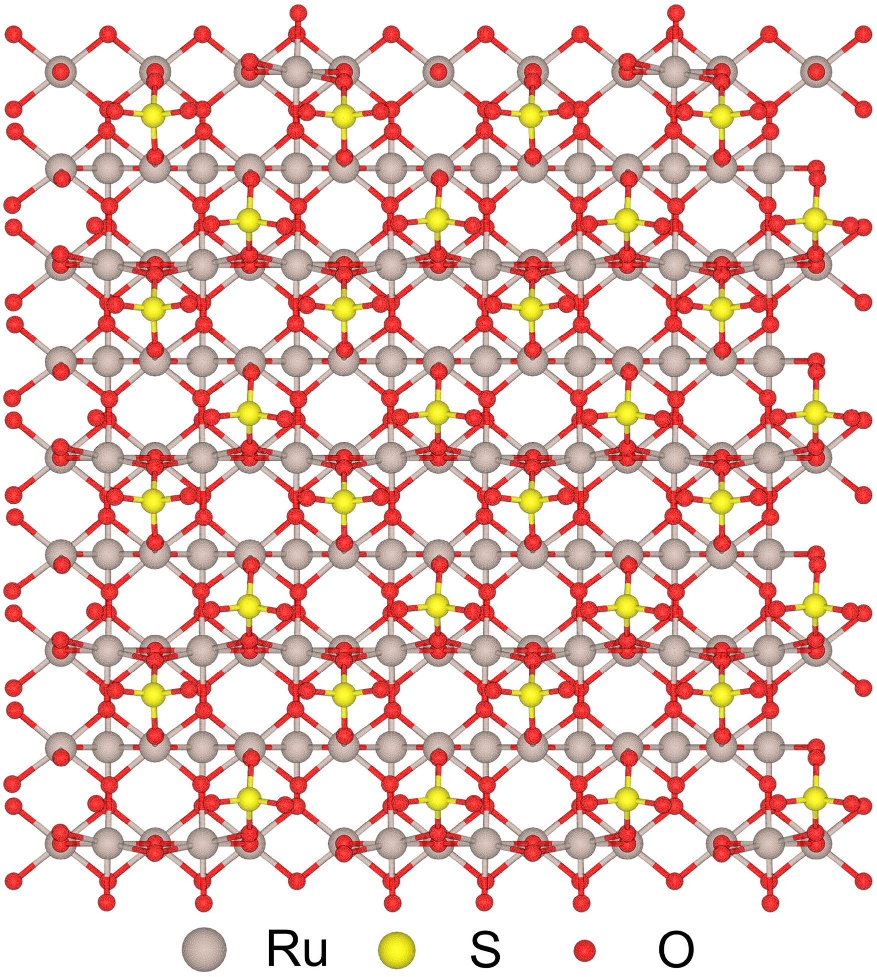

Supplementary Figure 3 Ball-and-stick model of sulfate bound with RuO_2 (110) plane.

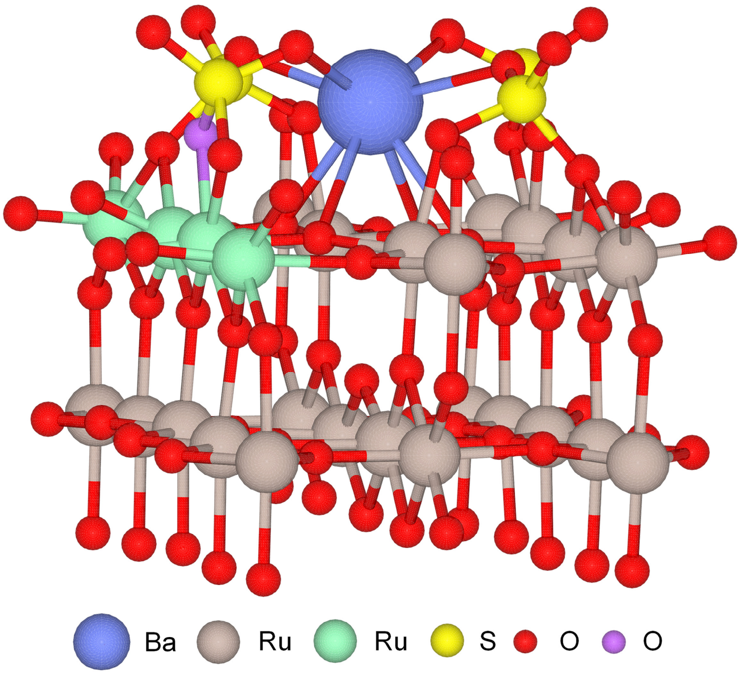

Supplementary Figure 4 Ball-and-stick model of Ba-anchored sulfate on the RuO_2 (110) plane. Of note, the green and purple balls represent Ru and O atoms, respectively, on the grain edges.

Supplementary Note 2: In this model, two O atoms of a sulfate bond to Ru atoms, and Ba atom bonds with one O atom of sulfate to fix the sulfate on the RuO_2 (110) plane. When calculating the binding energy of sulfate with other metal cations, Ba was replaced by other elements.

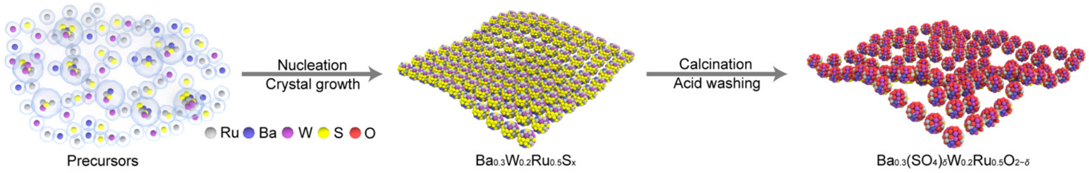

Supplementary Figure 5 Schematic illustrating the synthesis process of $Ba_{0.3}(SO_4)_{\delta}W_{0.2}Ru_{0.5}O_{2-\delta}$.

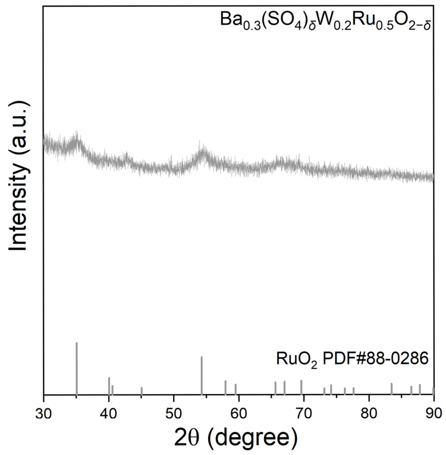

Supplementary Figure 6 XRD pattern of $Ba_{0.3}(SO_4)_{\delta}W_{0.2}Ru_{0.5}O_{2-\delta}$.

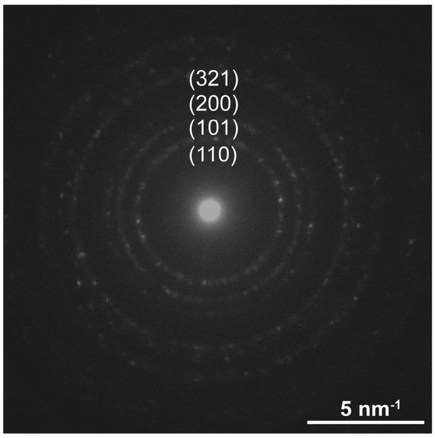

Supplementary Figure 7 SAED pattern of the $Ba_{0.3}(SO_4)_{\delta}W_{0.2}Ru_{0.5}O_{2-\delta}$.

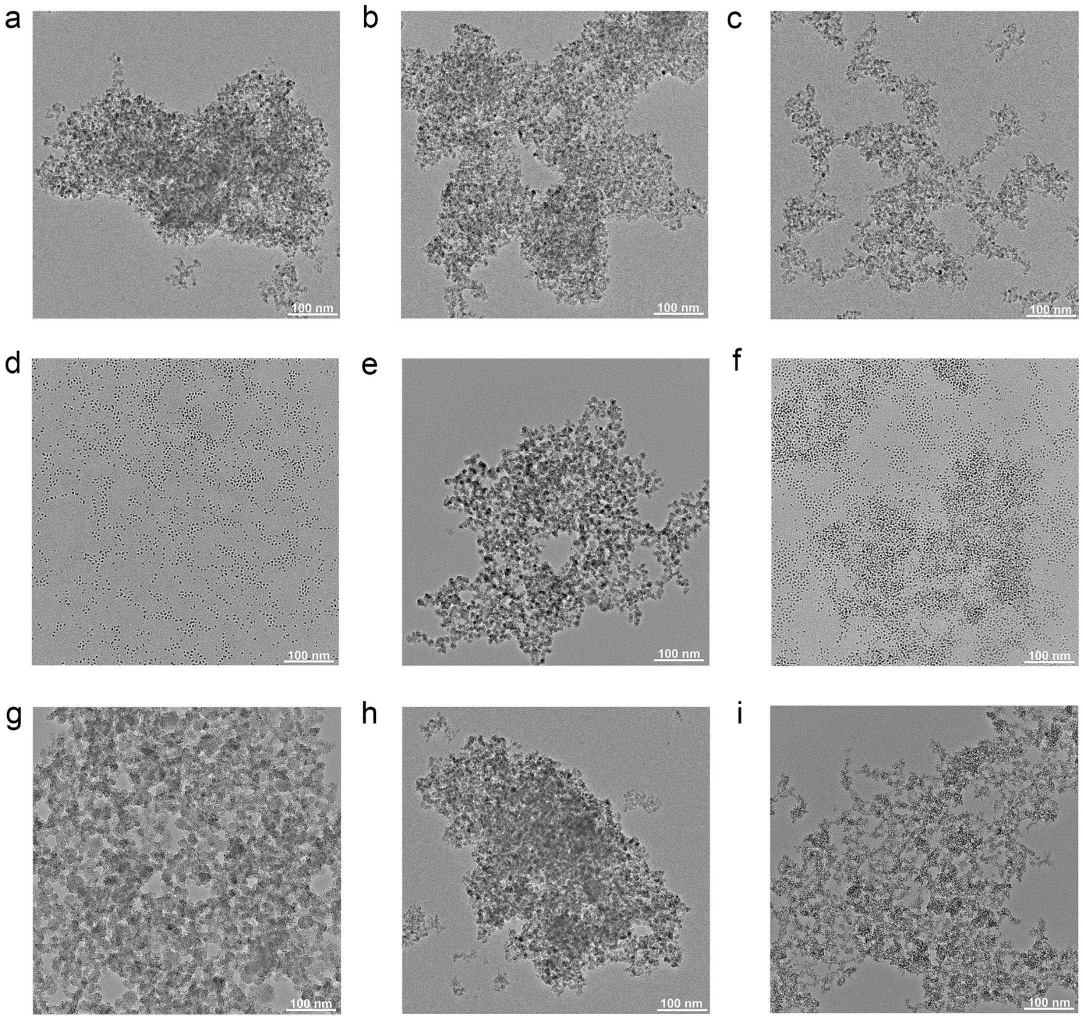

Supplementary Figure 8 a-i TEM images of (**a**) $Ba_{0.2}(SO_4)_{\delta}W_{0.1}Ru_{0.7}O_{2-\delta}$, (**b**) $Ba_{0.4}(SO_4)_{\delta}W_{0.2}Ru_{0.4}O_{2-\delta}$, and (**c**) $Ba_{0.4}(SO_4)_{\delta}W_{0.3}Ru_{0.3}O_{2-\delta}$, (**d**) as-prepared RuO_2, (**e**) $W_{0.3}Ru_{0.7}O_2$, (**f**) $Ba_{0.4}Ru_{0.6}O_2$, (**g**) $(SO_4)_{\delta}RuO_{2-\delta}$, and (**h**) $Ba_{0.4}(SO_4)_{\delta}Ru_{0.6}O_{2-\delta}$, and (**i**) $W_{0.3}(SO_4)_{\delta}Ru_{0.7}O_{2-\delta}$.

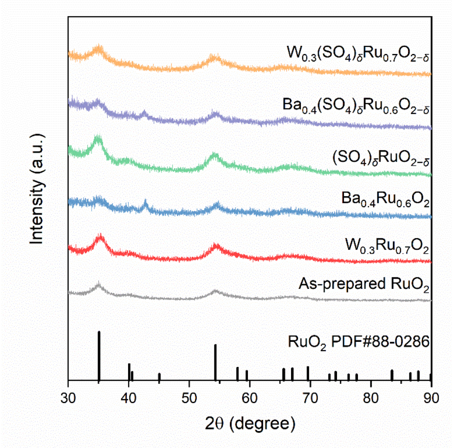

Supplementary Figure 9 XRD patterns of as-prepared RuO_2, $W_{0.3}Ru_{0.7}O_2$, $Ba_{0.4}Ru_{0.6}O_2$, $(SO_4)_\delta RuO_{2-\delta}$, $Ba_{0.4}(SO_4)_\delta Ru_{0.6}O_{2-\delta}$, and $W_{0.3}(SO_4)_\delta Ru_{0.7}O_{2-\delta}$.

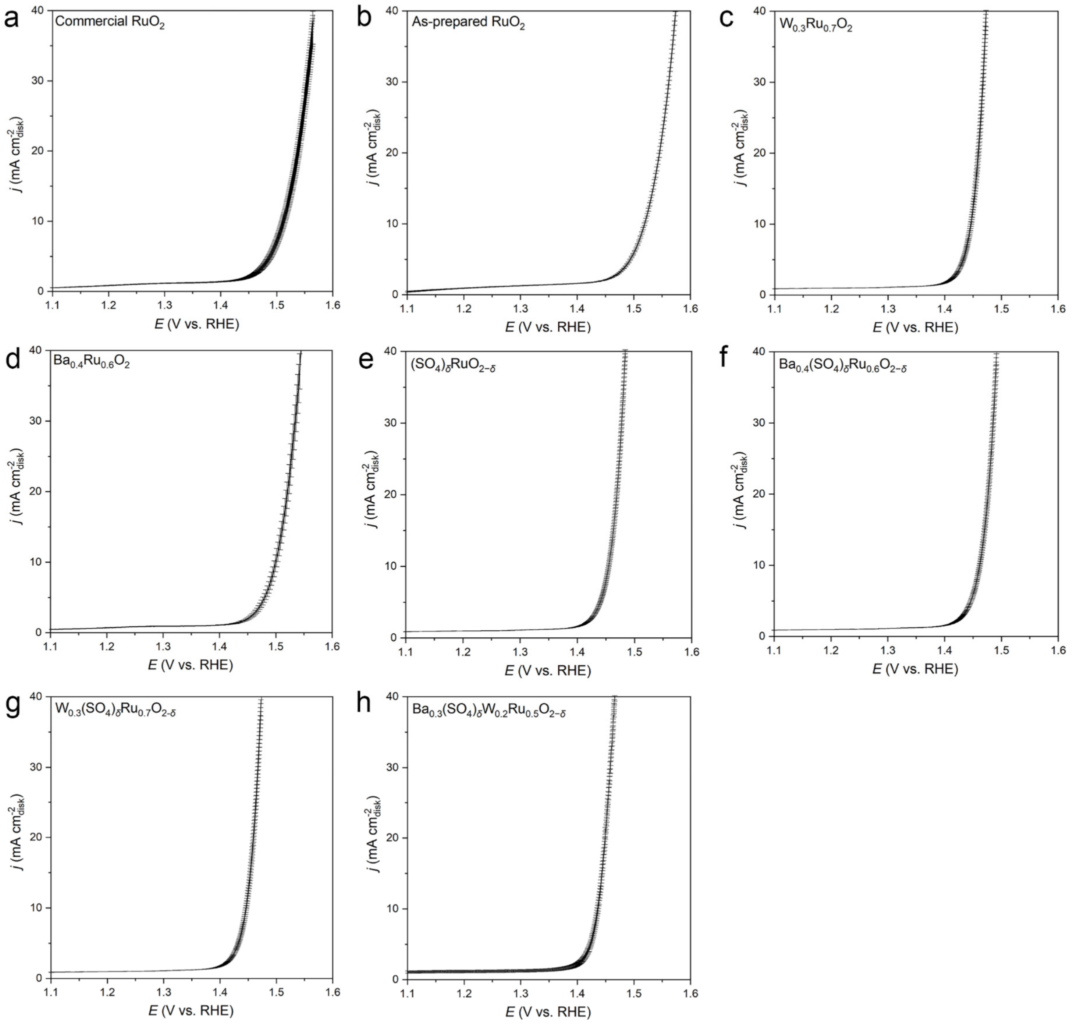

Supplementary Figure 10 a-h The mean OER polarization curves with error bars for (**a**) commercial RuO_2, (**b**) as-prepared RuO_2, (**c**) $W_{0.3}Ru_{0.7}O_2$, (**d**) $Ba_{0.4}Ru_{0.6}O_2$, (**e**) $(SO_4)_\delta RuO_{2-\delta}$, (**f**) $Ba_{0.4}(SO_4)_\delta Ru_{0.6}O_{2-\delta}$, (**g**) $W_{0.3}(SO_4)_\delta Ru_{0.7}O_{2-\delta}$, and (**h**) $Ba_{0.3}(SO_4)_\delta W_{0.2}Ru_{0.5}O_{2-\delta}$ based on three independent tests.

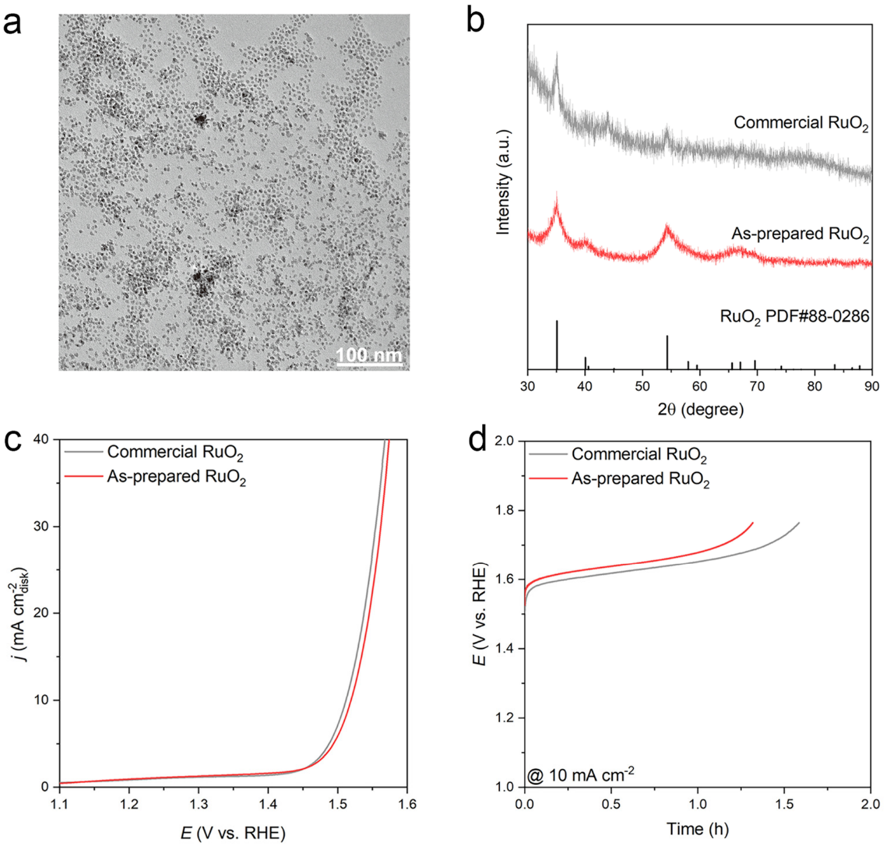

Supplementary Figure 11 a TEM images of commercial RuO_2. **b** XRD patterns of commercial RuO_2 and as-prepared RuO_2. **c** OER polarization curves of commercial RuO_2 and as-prepared RuO_2. **d** Chronopotentiogram of commercial RuO_2 and as-prepared RuO_2 at 10 mA cm^{-2} in 0.5 M H_2SO_4.

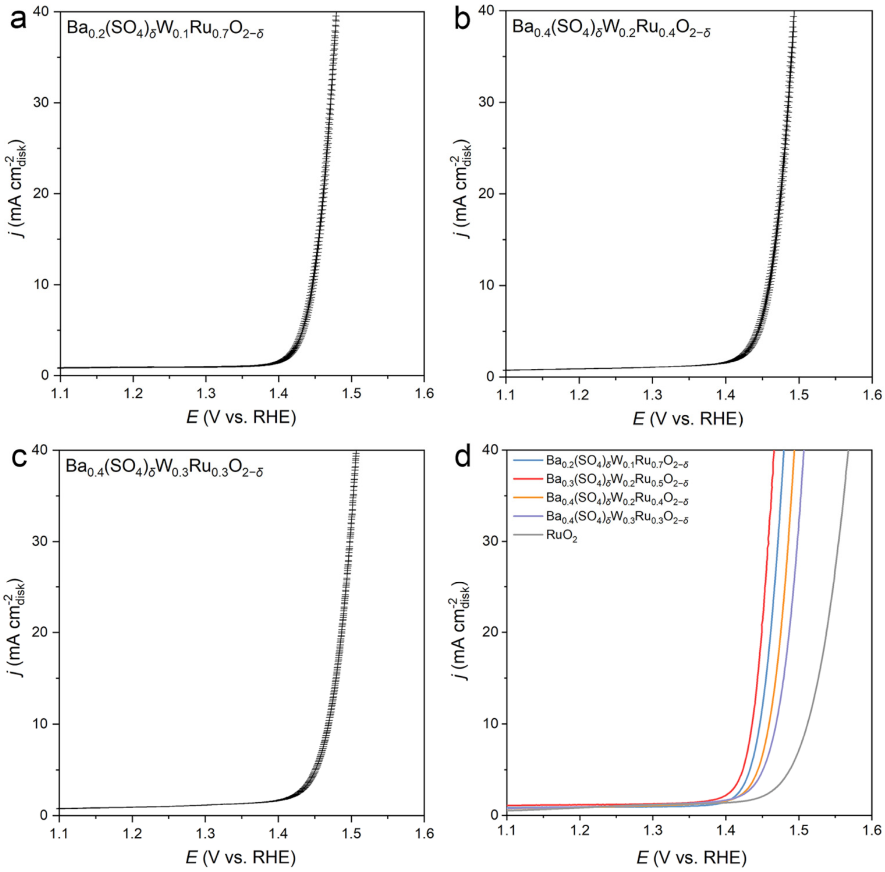

Supplementary Figure 12 a-c The mean OER polarization curves with error bars for (**a**) $Ba_{0.2}(SO_4)_\delta W_{0.1}Ru_{0.7}O_{2-\delta}$, (**b**) $Ba_{0.4}(SO_4)_\delta W_{0.2}Ru_{0.4}O_{2-\delta}$, (**c**) $Ba_{0.4}(SO_4)_\delta W_{0.3}Ru_{0.3}O_{2-\delta}$ based on three independent tests. **d** OER polarization curves of RuO_2, $Ba_{0.2}(SO_4)_\delta W_{0.1}Ru_{0.7}O_{2-\delta}$, $Ba_{0.3}(SO_4)_\delta W_{0.2}Ru_{0.5}O_{2-\delta}$, $Ba_{0.4}(SO_4)_\delta W_{0.2}Ru_{0.4}O_{2-\delta}$, and $Ba_{0.4}(SO_4)_\delta W_{0.3}Ru_{0.3}O_{2-\delta}$.

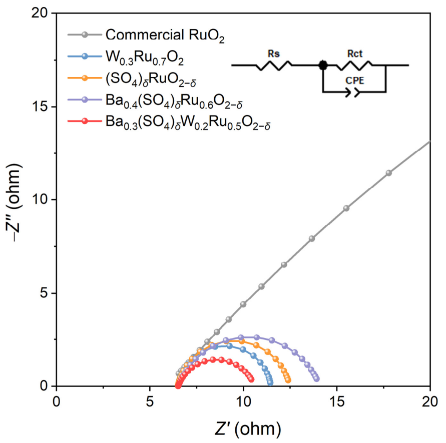

Supplementary Figure 13 Nyquist plots of commercial RuO_2, $W_{0.3}Ru_{0.7}O_2$, $(SO_4)_\delta RuO_{2-\delta}$, $Ba_{0.4}(SO_4)_\delta Ru_{0.6}O_{2-\delta}$, and $Ba_{0.3}(SO_4)_\delta W_{0.2}Ru_{0.5}O_{2-\delta}$.

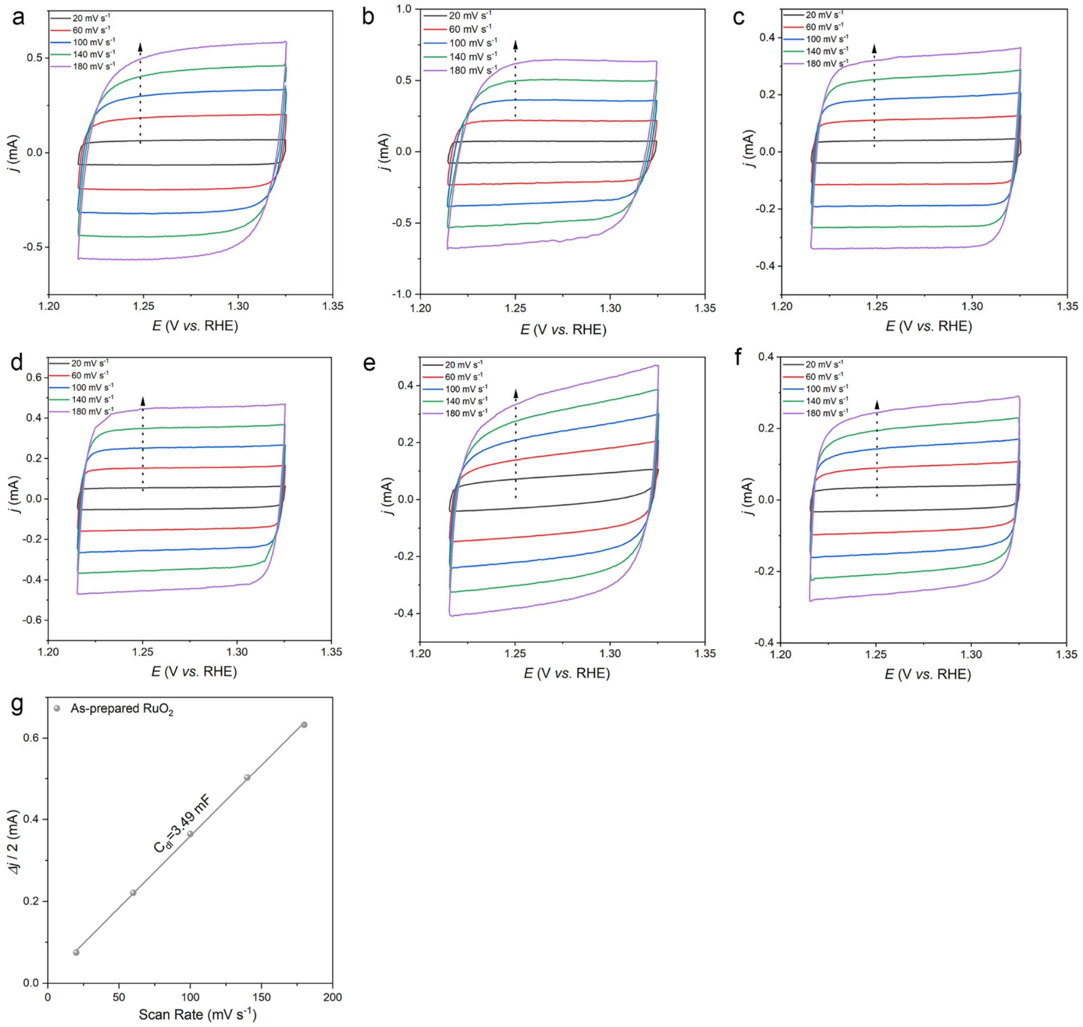

Supplementary Figure 14 a-f Cyclic voltammograms of (**a**) commercial RuO_2, (**b**) as-prepared RuO_2. (**c**) $W_{0.3}Ru_{0.7}O_2$, (**d**) $(SO_4)_\delta RuO_{2-\delta}$, (**e**) $Ba_{0.4}(SO_4)_\delta Ru_{0.6}O_{2-\delta}$, and (**f**) $Ba_{0.3}(SO_4)_\delta W_{0.2}Ru_{0.5}O_{2-\delta}$ at scan rates of 20, 60, 100, 140, and 180 mV s^{-1}. **g** C_{dl} plots of as-prepared RuO_2 at 1.25 V.

Supplementary Note 3: For particles, both the specific surface area and the ECSA increase with the decrease of the size. In comparison to commercial RuO_2, the size of as-prepared RuO_2 is smaller, resulting in a larger C_{dl} and ECSA (Supplementary Fig. 14g). After the formation of Ru alloys with Ba or W, the particle size becomes larger, leading to a smaller ECSA. Additionally, Ru coordinates with sulfate to easily form a large nanosheet-like structure, which can also reduce the ECSA.

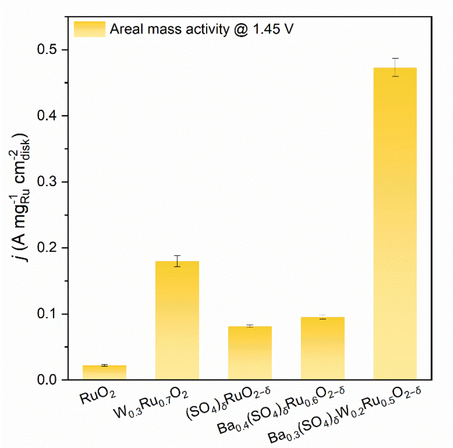

Supplementary Figure 15 Areal mass activity at 1.45 V of commercial RuO_2, $W_{0.3}Ru_{0.7}O_2$, $(SO_4)_\delta RuO_{2-\delta}$, $Ba_{0.4}(SO_4)_\delta Ru_{0.6}O_{2-\delta}$, and $Ba_{0.3}(SO_4)_\delta W_{0.2}Ru_{0.5}O_{2-\delta}$.

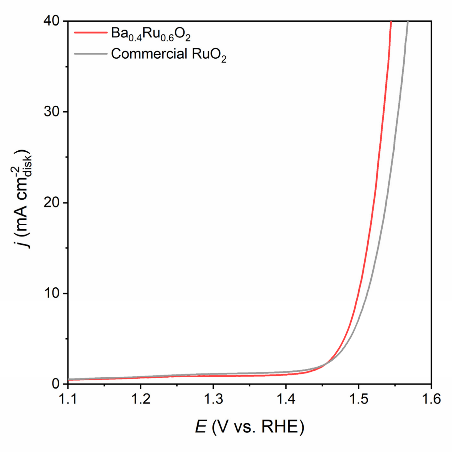

Supplementary Figure 16 OER polarization curves of $Ba_{0.4}Ru_{0.6}O_2$ and commercial RuO_2.

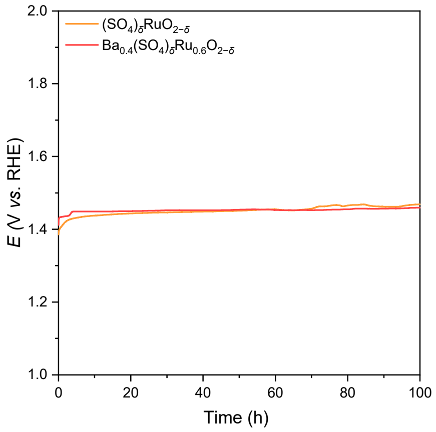

Supplementary Figure 17 Chronopotentiogram of $(SO_4)_\delta RuO_{2-\delta}$ and $Ba_{0.4}(SO_4)_\delta Ru_{0.6}O_{2-\delta}$ at 10 mA cm^{-2} in 0.1 M $HClO_4$.

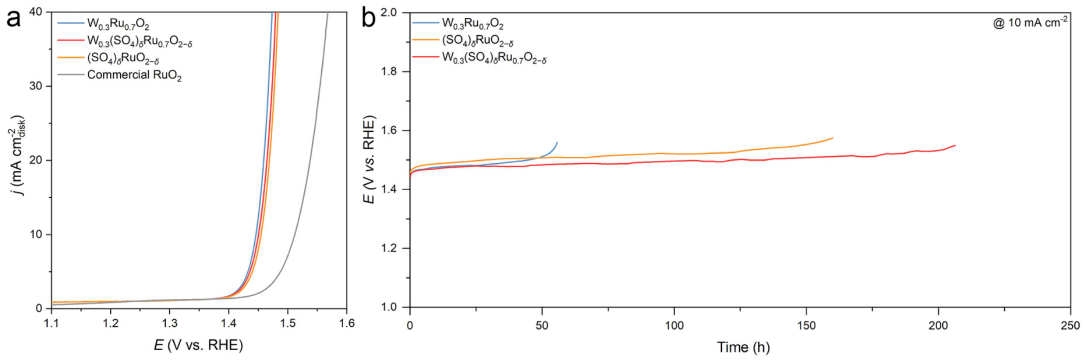

Supplementary Figure 18 a OER polarization curves of commercial RuO_2, $W_{0.3}Ru_{0.7}O_2$, $(SO_4)_\delta RuO_{2-\delta}$, and $W_{0.3}(SO_4)_\delta Ru_{0.7}O_{2-\delta}$. **b** Chronopotentiogram of $W_{0.3}Ru_{0.7}O_2$, $(SO_4)_\delta RuO_{2-\delta}$, and $W_{0.3}(SO_4)_\delta Ru_{0.7}O_{2-\delta}$ at 10 mA cm^{-2} in 0.5 M H_2SO_4.

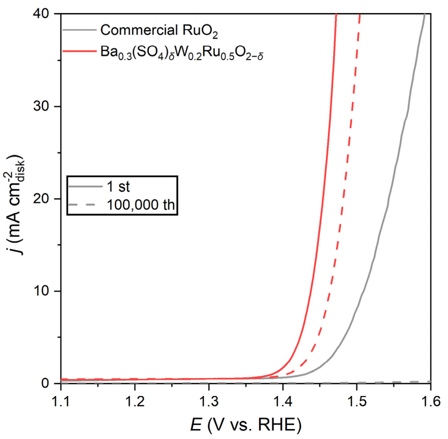

Supplementary Figure 19 OER polarization curves of commercial RuO_2 and $Ba_{0.3}(SO_4)_\delta W_{0.2}Ru_{0.5}O_{2-\delta}$ before and after 100,000 cycles.

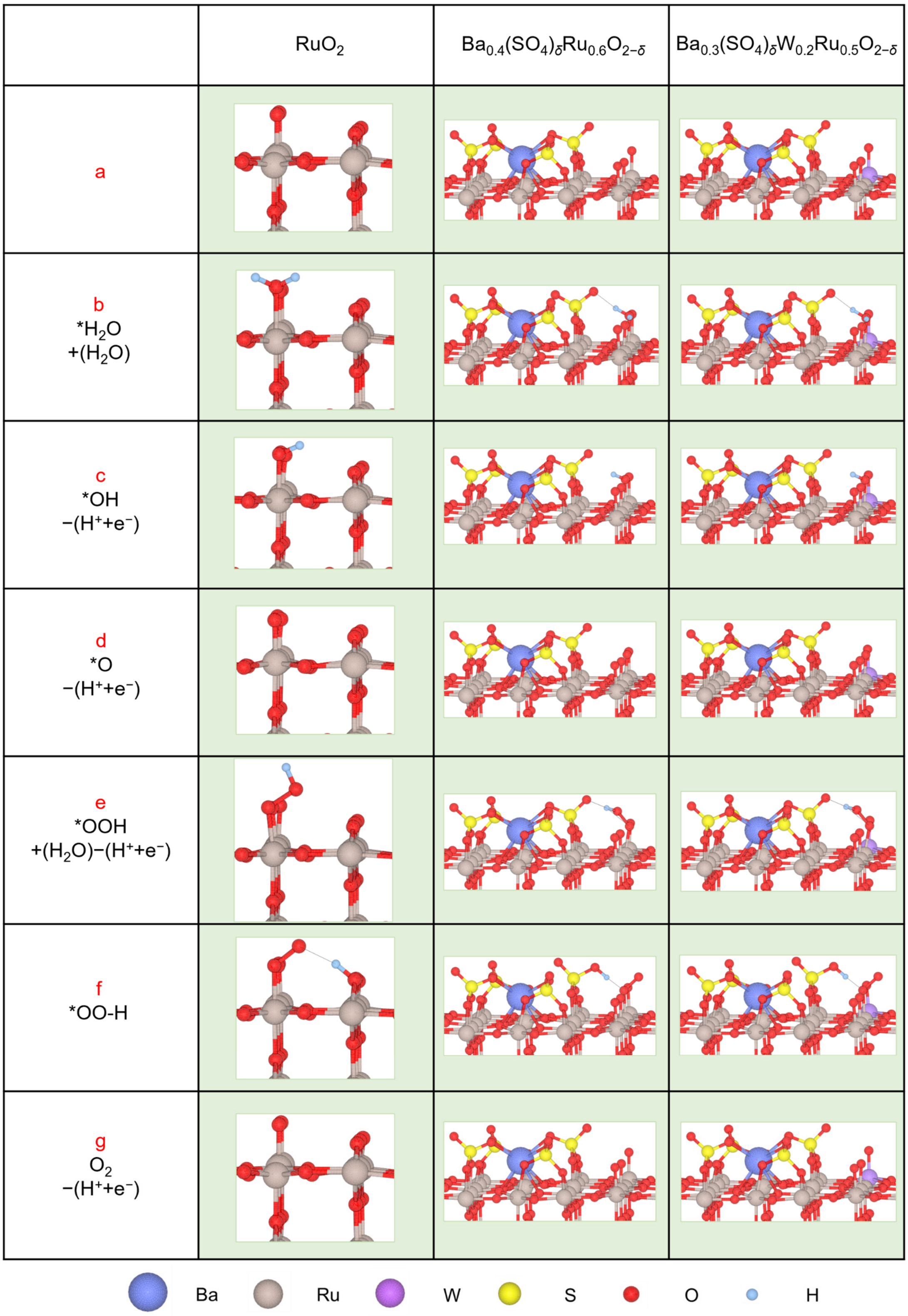

Supplementary Figure 20 Atomic structure of catalysts and intermediates during the OER process.

Supplementary Note 4: For RuO_2, an H_2O molecule is adsorbed on the 1f-cus Ru site of the RuO_2(110) plane (a to b) and undergoes continuous deprotonation, leading to the formation of *OH and *O on the same site (b to c, c to d). Subsequently, a second H_2O molecule is adsorbed onto the *O and deprotonates,

resulting in the formation of *OOH (d to e). Afterward, the *OOH donates a proton to an adjacent O site to form *OO-H (e to f). Finally, the *OO-H undergoes deprotonation, leading to the release of O_2 (f to g).

For $Ba_{0.4}(SO_4)_{\delta}Ru_{0.6}O_{2-\delta}$, the OER process is similar to RuO_2. An H_2O molecule is adsorbed on the 1f-cus Ru site (a to b), and deprotonated to form *OH and *O on this site (b to c, c to d). Then, a second H_2O molecule is adsorbed on *O and deprotonates to form *OOH (d to e). Here, the *OOH donates a proton to an adjacent O site, which belongs to a sulfate to form *OO-H (e to f). Finally, the *OO-H is deprotonated and an O_2 is released (f to g).

For $Ba_{0.3}(SO_4)_{\delta}W_{0.2}Ru_{0.5}O_{2-\delta}$, the OER process is identical to that of $Ba_{0.4}(SO_4)_{\delta}Ru_{0.6}O_{2-\delta}$. In $Ba_{0.3}(SO_4)_{\delta}W_{0.2}Ru_{0.5}O_{2-\delta}$, a Ru atom adjacent to the active site in the crystal is replaced with a W atom, forming Ru-O-W. This substitution allows W to modulate the electronic structure of the 1f-cus-Ru site, thereby adjusting the adsorption energy of the reaction intermediates.

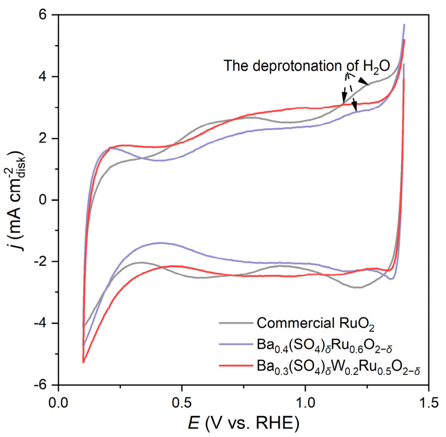

Supplementary Figure 21 Cyclic voltammograms of commercial RuO_2, $Ba_{0.4}(SO_4)_{\delta}Ru_{0.6}O_{2-\delta}$, and $Ba_{0.3}(SO_4)_{\delta}W_{0.2}Ru_{0.5}O_{2-\delta}$ in Ar-saturated 0.5 M H_2SO_4 at a scan rate of 50 mV s^{-1}.

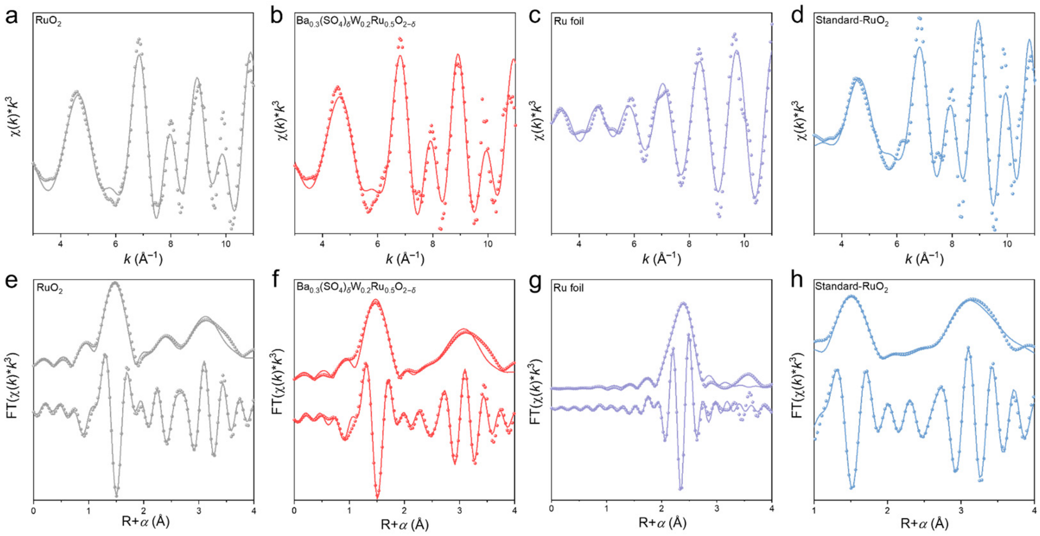

Supplementary Figure 22 a-d Ru K-edge EXAFS (points) and the curvefit (line) for (**a**) RuO_2, (**b**) $Ba_{0.3}(SO_4)_{\delta}W_{0.2}Ru_{0.5}O_{2-\delta}$, (**c**) Ru foil, and (**d**) standard-RuO_2, shown in k^3weighted *k*-space. **e-h**, Ru K-edge EXAFS (points) and the curvefit (line) for (**e**) RuO_2, (**f**) $Ba_{0.3}(SO_4)_{\delta}W_{0.2}Ru_{0.5}O_{2-\delta}$, (**g**) Ru foil, and (**h**) standard-RuO_2, shown in *R*-space (FT magnitude and imaginary component). The data are k^3-weighted and not phase-corrected.

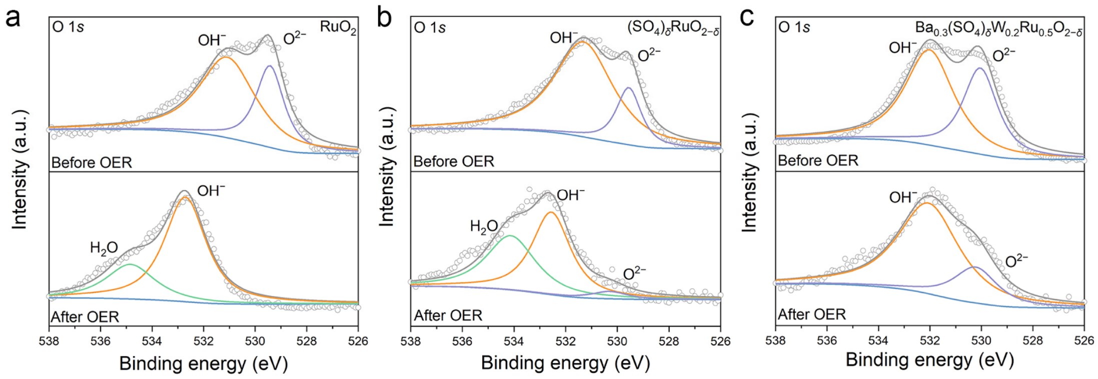

Supplementary Figure 23 a-c High-resolution O 1*s* XPS spectra of (**a**) RuO_2, (**b**) $(SO_4)_\delta RuO_{2-\delta}$, and (**c**) $Ba_{0.3}(SO_4)_\delta W_{0.2}Ru_{0.5}O_{2-\delta}$ before and after OER.

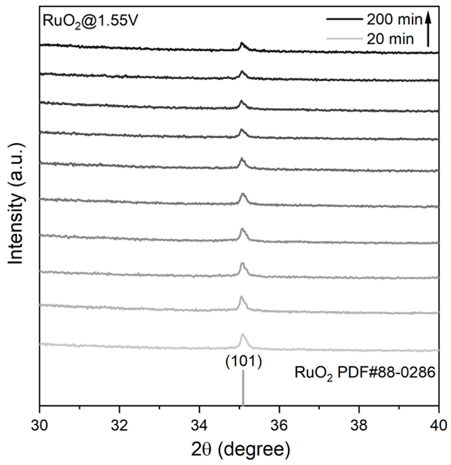

Supplementary Figure 24 Time-resolved *in-situ* XRD patterns of RuO_2 during 200 min acidic OER at 1.55 V.

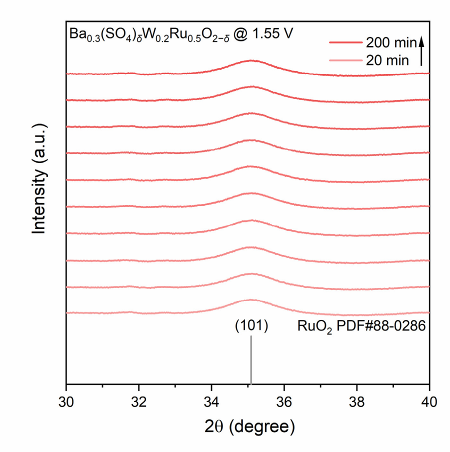

Supplementary Figure 25 Time-resolved *in-situ* XRD patterns of $Ba_{0.3}(SO_4)_{\delta}W_{0.2}Ru_{0.5}O_{2-\delta}$ during 200 min acidic OER at 1.55 V.

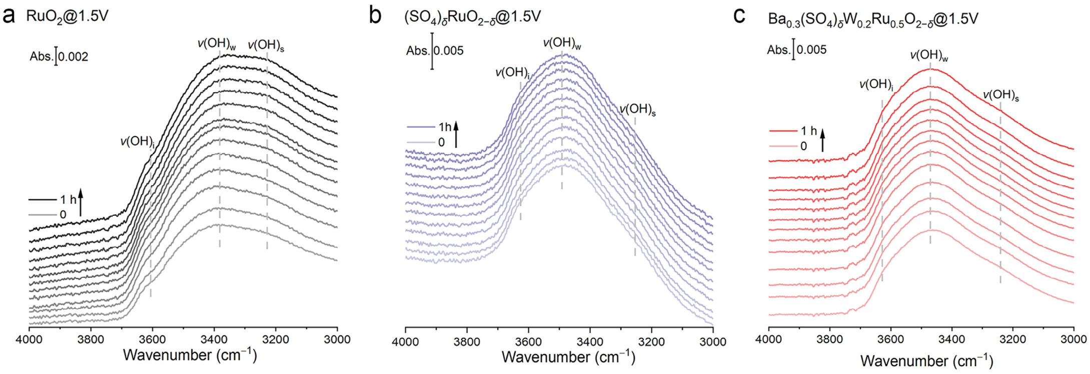

Supplementary Figure 26 a-c Time-resolved *in-situ* ATR-SEIRAS spectra of (**a**) RuO_2, (**b**) $(SO_4)_\delta RuO_{2-\delta}$, and (**c**) $Ba_{0.3}(SO_4)_\delta W_{0.2}Ru_{0.5}O_{2-\delta}$ over the course of 1 h acidic OER at 1.5 V.

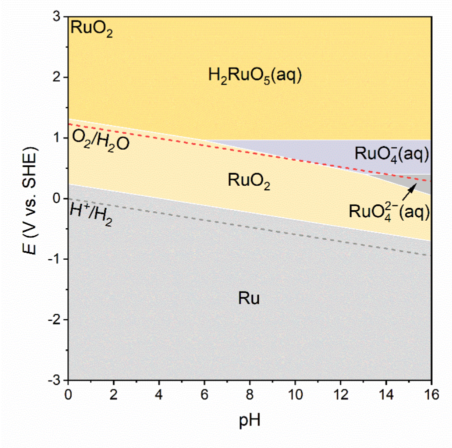

Supplementary Figure 27 Ru Pourbaix diagram for RuO_2 generated with an aqueous ion concentration of 10^{-6} M at 25 °C.

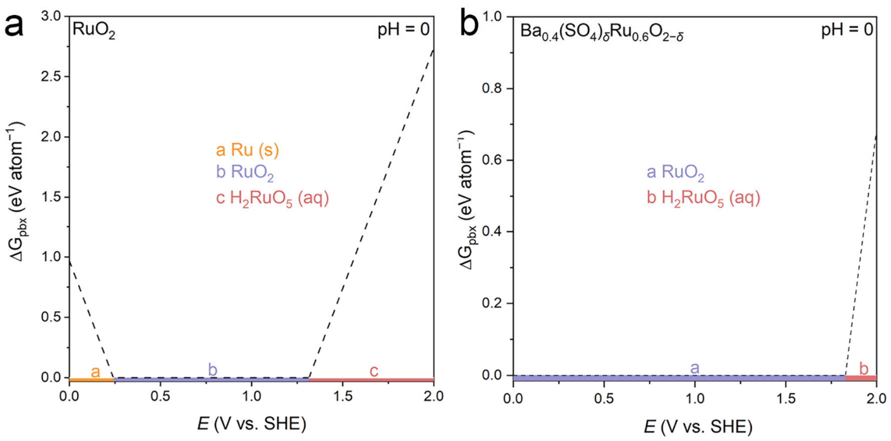

Supplementary Figure 28 a,b Pourbaix decomposition free energy (ΔG_{pbx}) of (**a**) RuO_2 and (**b**) $Ba_{0.4}(SO_4)_\delta Ru_{0.6}O_{2-\delta}$ at potentials between 0 and 2.0 V.

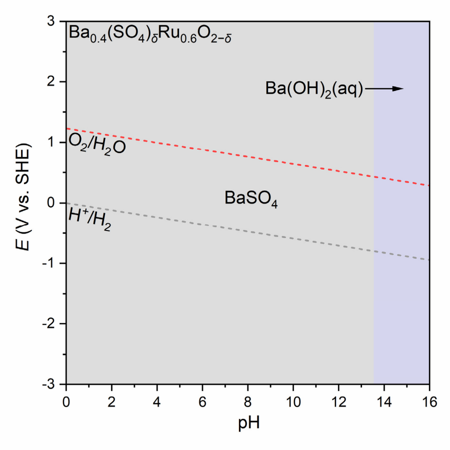

Supplementary Figure 29 Ba Pourbaix diagram for $Ba_{0.4}(SO_4)_{\delta}Ru_{0.6}O_{2-\delta}$ generated with an aqueous ion concentration of 10^{-6} M at 25 °C.

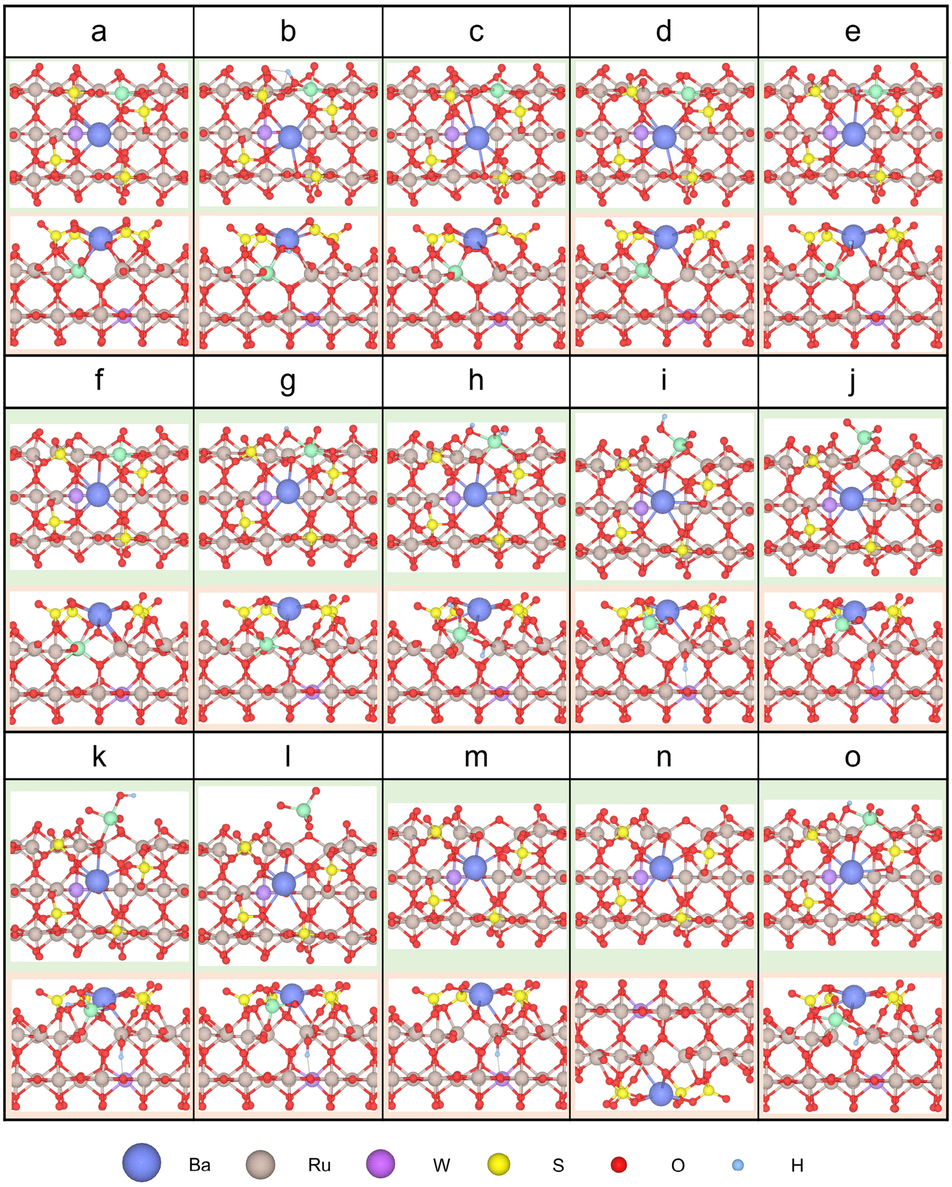

Supplementary Figure 30 Atomic structures of catalysts and intermediates during Ru dissolution on $Ba_{0.3}(SO_4)_\delta W_{0.2}Ru_{0.5}O_{2-\delta}$.

Supplementary Note 5: In the most possible thermodynamic deactivation pathway of $Ba_{0.3}(SO_4)_\delta W_{0.2}Ru_{0.5}O_{2-\delta}$, the adsorption and deprotonation of water are optimized on the most energetically favorable sites. Benefiting from the protection of Ba-anchored sulfate, the deactivation of $Ba_{0.3}(SO_4)_\delta W_{0.2}Ru_{0.5}O_{2-\delta}$ needs to overcome two positive free energy difference in the steps from *a* to *b* and *k* to *l*, respectively, while that of RuO_2 is almost spontaneous – that means, it is more difficult and sluggish for $Ba_{0.3}(SO_4)_\delta W_{0.2}Ru_{0.5}O_{2-\delta}$ to deactivate than RuO_2.

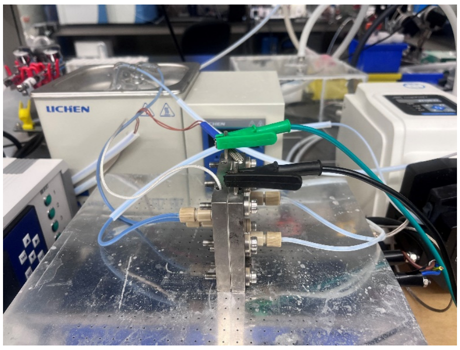

Supplementary Figure 31 Photograph of the PEMWE.

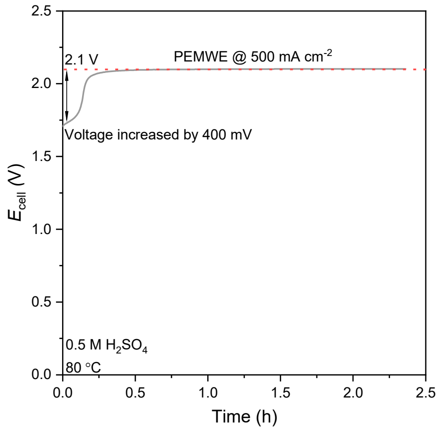

Supplementary Figure 32 Chronopotentiogram of commercial RuO_2-based PEMWE operated at 500 mA cm^{-2} in 0.5 M H_2SO_4 under 80 °C.

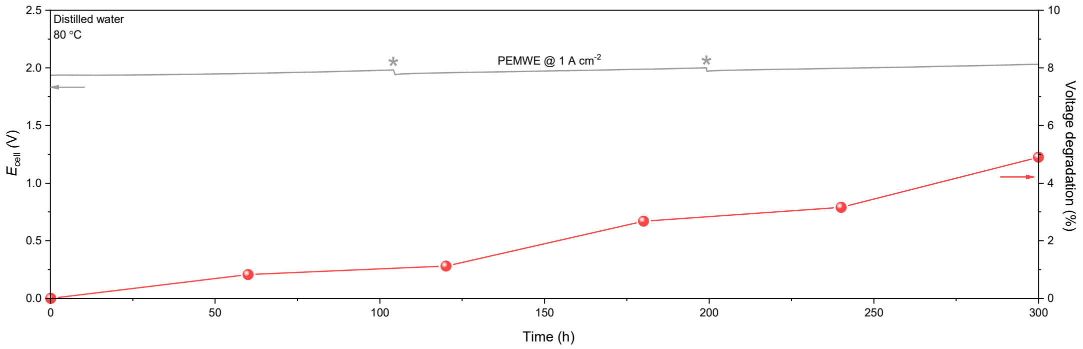

Supplementary Figure 33 Chronopotentiogram and voltage degradation of $Ba_{0.3}(SO_4)_{\delta}W_{0.2}Ru_{0.5}O_{2-\delta}$-based PEMWE operated at 1 A cm^{-2} in distilled water under 80 °C. Of note, the gray asterisks represent the replenishment of fresh electrolytes, and the difference in cell voltage between 0.5 M H_2SO_4 and distilled water results from the impedance difference between the electrolyte and the MEA.

Supplementary Tables

Supplementary Table 1. The binding energy of various metal cations with sulfate on the surface of RuO_2 (110) plane.

Metal	Binding Energy (eV)	Metal	Binding Energy (eV)	Metal	Binding Energy (eV)	Metal	Binding Energy (eV)
Au	−3.26	Te	−7.01	Al	−8.65	Re	−14.5
Ag	−4.03	Ni	−7.11	Fe	−8.82	V	−14.6
Cd	−4.8	Ge	−7.23	Mg	−8.94	Sc	−14.8
Se	−5.15	Pt	−7.33	Ir	−9.5	Ti	−15.3
Ga	−5.17	Sn	−7.36	Be	−10.2	Pr	−15.6
Zn	−5.49	Sb	−7.51	Sr	−10.4	Y	−15.9
Cu	−5.63	Pb	−7.56	Ca	−10.5	La	−16
Pd	−5.81	In	−7.78	Ru	−10.5	Mo	−16
Na	−5.92	Co	−7.85	Ba	−10.8	Ce	−17.1
K	−6.04	Bi	−7.93	Eu	−11.4	Gd	−17.1
Li	−6.52	Rh	−8.01	Cr	−12.1	Zr	−18.6
As	−6.87	Mn	−8.6	Nd	−14.5	Nb	−19
W	−19.19	Hf	−19.3	Ta	−20.2		

Supplementary Table 2. OER performances of the Ru-based catalysts in this work.

Catalysts	Loading (μg cm^{-2})	ECSA (cm^2_{oxide})	Overpotential (V) @ 10 mA cm^{-2}	j_m @ 1.45 V (A mg^{-1}_{oxide})	j_s @ 1.45 V (mA cm^{-2}_{oxide})
RuO_2	125	83.4	282	0.003	0.005
$W_{0.3}Ru_{0.7}O_2$	125	52.3	216	0.020	0.047
$(SO_4)_{\delta}RuO_{2-\delta}$	125	71.1	225	0.013	0.023
$Ba_{0.4}(SO_4)_{\delta}Ru_{0.6}O_{2-\delta}$	125	54.6	232	0.009	0.022
$Ba_{0.3}(SO_4)_{\delta}W_{0.2}Ru_{0.5}O_{2-\delta}$	125	39.7	206	0.034	0.105

Supplementary Table 3. The amount of dissolved Ru, Ba and W for various catalysts after OER.

Catalyst	Ru Loading (μg)	Ba Loading (μg)	W Loading (μg)	Dissolved Ru (μg)	Dissolved Ba (μg)	Dissolved W (μg)
RuO_2	95.05			33.84		
$W_{0.3}Ru_{0.7}O_2$	70.12		55.24	16.90		7.23
$(SO_4)_\delta RuO_{2-\delta}$	100.25			19.25		
$Ba_{0.4}(SO_4)_\delta Ru_{0.6}O_{2-\delta}$	62.54	46.93		7.19	1.59	
$Ba_{0.3}(SO_4)_\delta W_{0.2}Ru_{0.5}O_{2-\delta}$	45.12	33.82	34.76	3.20	0.81	3.22

Supplementary Table 4. The stability number (S-number) of various catalysts after OER stability test.

Catalyst	Ru loss (%)	Ba loss (%)	W loss (%)	Reaction time (h)	S-number
RuO_2	35.6			1.5	418
$W_{0.3}Ru_{0.7}O_2$	24.11		13.08	55	30682
$(SO_4)_\delta RuO_{2-\delta}$	19.19			160	78360
$Ba_{0.4}(SO_4)_\delta Ru_{0.6}O_{2-\delta}$	11.49	3.39		316	414346
$Ba_{0.3}(SO_4)_\delta W_{0.2}Ru_{0.5}O_{2-\delta}$	7.09	2.40	9.26	1000	2946151

Supplementary Table 5. Summary of the reported Ru-based OER catalysts under acidic conditions.

Catalyst	Loading (μg cm^{-2})	Electrolyte	Overpotential @10 mA cm^{-2} (mV)	Overpotential increase @10 mA cm^{-2}	Ref.
Ni-RuO_2	400	0.1 M $HClO_4$	214	Stable for 200 h	1
Co-RuIr	50	0.1 M $HClO_4$	235	Stable for 25 h	2
Faceted Ru	127	0.1 M $HClO_4$	180	85 mV for 4 h	3
Ru_1-Pt_3Cu	21	0.1 M $HClO_4$	220	30 mV for 28 h	4
$Bi_xEr_{2-x}Ru_2O_7$	830	0.1 M $HClO_4$	180	Stable for 100 h	5
Cu-doped RuO_2	275	0.5 M H_2SO_4	188	83 mV for 8 h	6
$CaCu_3Ru_4O_{12}$	250	0.5 M H_2SO_4	171	21 mV for 24 h	7
$Cr_{0.6}Ru_{0.4}O_2$	283	0.5 M H_2SO_4	178	Stable for 10 h	8
$W_{0.2}Er_{0.1}Ru_{0.7}O_{2-\delta}$	330	0.5 M H_2SO_4	168	83 mV for 500 h	9
$Li_{0.52}RuO_2$	637	0.5 M H_2SO_4	156	Stable for 70 h	10
$Y_{1.7}Sr_{0.3}Ru_2O_7$	71	0.5 M H_2SO_4	264	Stable for 28 h	11
$RuNi_2$©G-250	320	0.5 M H_2SO_4	227	Stable for 3 h	12
$Ba_{0.3}(SO_4)_\delta W_{0.2}Ru_{0.5}O_{2-\delta}$	125	0.5 M H_2SO_4	206	43 mV for 1,000 h	This work

Supplementary Table 6. Curvefit parameters for Ru K-edge EXAFS for various samples (S_0^2=0.9).

Catalyst	Path	R(Å)[a]	N[b]	σ^2 (10^{-3}Å^2)[c]	ΔE_0 (eV)[d]	R factor
RuO_2	Ru-O	1.97	4.4	2.4	−1.32	0.016
	Ru-Ru	2.7	1	1.2		
	Ru-O	3.16	2.5	3.5		
$Ba_{0.3}(SO_4)_\delta W_{0.2}Ru_{0.5}O_{2-\delta}$	Ru-O	1.97	4	0.6	−1.71	0.019
	Ru-Ru	2.69	1	4.2		
	Ru-O	3.15	2	11.4		
Ru foil	Ru-Ru	2.67	12	3.6	3.59	0.013
Standard RuO_2	Ru-O	1.96	6	−0.3	−0.32	0.018
	Ru-Ru	2.66	6	−1.5		
	Ru-O	3.17	2	4.9		

[a]*R*: Bond distance; [b]*N*: coordination numbers; [c]σ^2: Debye-Waller factors; [d]ΔE_0: the inner potential correction; S_0^2: the amplitude reduction factor, which was set to 0.9 in the fittings; R factor: goodness of fit.

Supplementary Table 7. Free energies of formation for DFT-computed phases in Pourbaix analysis.

RuO_2		$Ba_{0.4}(SO_4)_{\delta}Ru_{0.6}O_{2-\delta}$			
Ru	0	Ru	0	Ba	0
RuO_2	−3.95	RuO_2	−6.00	Ba^{2+}(aq)	−6.17
RuO_4^{2-}(aq)	−4.98	RuO_4^{2-}(aq)	−4.98	$BaOH^{+}$(aq)	−7.83
RuO_4^{-}(aq)	−4.58	RuO_4^{-}(aq)	−4.58	SO_4^{2-} (aq)	−8.43
H_2RuO_5(aq)	−6.07	H_2RuO_5(aq)	−6.07	$BaSO_4$	−14.60

Supplementary Table 8. Summary of the reported PEMWE stability using different Ru-based OER catalysts.

Anode catalyst	Mass loading (mg cm^{-2})	PEM	Cell temperature (°C)	Electrolyte	Current density (A cm^{-2})	Cell voltage increase	Ref.
PtCo–RuO_2/C	2.5	N212	80	Distilled water	1	11 mV for 24 h	13
RuO_2/SnO_2	3	N115	30	Distilled water	0.25	~ 400 mV for 235 h	14
RuO_2 NS	1.2	N212	90	Distilled water	1	Stable for 10 h	15
Ni-RuO_2	~3.1	N117	Room temperature	0.1 M $HClO_4$	0.2	Stable for 1,000 h	1
$W_{0.2}Er_{0.1}Ru_{0.7}O_{2-\delta}$	Not given	N117	Room temperature	0.5 M H_2SO_4	0.1	~ 100 mV for 120 h	9
$Ba_{0.3}(SO_4)_\delta W_{0.2}Ru_{0.5}O_{2-\delta}$	3	N115	80	0.5 M H_2SO_4	0.5	108 mV for 300 h	This work

Supplementary References

1 Wu, Z. Y. *et al.* Non-iridium-based electrocatalyst for durable acidic oxygen evolution reaction in proton exchange membrane water electrolysis. *Nat. Mater.* **22**, 100-108 (2023).

2 Shan, J., Ling, T., Davey, K., Zheng, Y. & Qiao, S. Z. Transition-metal-doped RuIr bifunctional nanocrystals for overall water splitting in acidic environments. *Adv. Mater.* **31**, e1900510 (2019).

3 Poerwoprajitno, A. R. *et al.* Formation of branched ruthenium nanoparticles for improved electrocatalysis of oxygen evolution reaction. *Small* **15**, e1804577 (2019).

4 Yao, Y. *et al.* Engineering the electronic structure of single atom Ru sites via compressive strain boosts acidic water oxidation electrocatalysis. *Nat. Catal.* **2**, 304-313 (2019).

5 Zhou, G. *et al.* Spin-related symmetry breaking induced by half-disordered hybridization in $Bi_xEr_{2-x}Ru_2O_7$ pyrochlores for acidic oxygen evolution. *Nat. Commun.* **13**, 4106 (2022).

6 Su, J. *et al.* Assembling ultrasmall copper-doped ruthenium oxide nanocrystals into hollow porous polyhedra: highly robust electrocatalysts for oxygen evolution in acidic media. *Adv. Mater*. **30**, 1801351 (2018).

7 Miao, X. *et al.* Quadruple perovskite ruthenate as a highly efficient catalyst for acidic water oxidation. *Nat. Commun.* **10**, 3809 (2019).

8 Lin, Y. *et al.* Chromium-ruthenium oxide solid solution electrocatalyst for highly efficient oxygen evolution reaction in acidic media. *Nat. Commun.* **10**, 162 (2019).

9 Hao, S. *et al.* Dopants fixation of ruthenium for boosting acidic oxygen evolution stability and activity. *Nat. Commun.* **11**, 5368 (2020).

10 Qin, Y. *et al.* RuO_2 electronic structure and lattice strain dual engineering for enhanced acidic oxygen evolution reaction performance. *Nat. Commun.* **13**, 3784 (2022).

11 Zhang, N. *et al.* Metal substitution steering electron correlations in pyrochlore ruthenates for efficient acidic water oxidation. *ACS Nano* **15**, 8537-8548 (2021).

12 Cui, X. *et al.* Robust interface Ru centers for high-performance acidic oxygen evolution. *Adv. Mater.* **32**, e1908126 (2020).

13 Jin, H. *et al.* Safeguarding the RuO_2 phase against lattice oxygen oxidation during acidic water electrooxidation. *Energy Environ. Sci.* **15**, 1119 (2022).

14 Lim, J. Y. *et al.* Highly stable RuO_2/SnO_2 nanocomposites as anode electrocatalysts in a PEM water electrolysis cell. *Int. J. Energy Res.* **38**, 875-883 (2014).

15 Huang, H. *et al.* Structure engineering defective and mass transfer-enhanced RuO_2 nanosheets for proton exchange membrane water electrolyzer. *Nano Energy* **88**, 106276 (2021).
